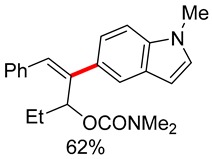# Indolylboronic Acids: Preparation and Applications

**DOI:** 10.3390/molecules24193523

**Published:** 2019-09-28

**Authors:** Marek Čubiňák, Tereza Edlová, Peter Polák, Tomáš Tobrman

**Affiliations:** Department of Organic Chemistry, University of Chemistry and Technology Prague, Technická 5, 166 28 Prague 6, Czech Republic; marek.cubinak@vscht.cz (M.Č.); tereza.edlova@vscht.cz (T.E.); peter.polak@vscht.cz (P.P.)

**Keywords:** indole, boronic acid, C−H borylation, Suzuki reaction, multicomponent reaction, cross-coupling

## Abstract

Indole derivatives are associated with a variety of both biological activities and applications in the field of material chemistry. A number of different strategies for synthesizing substituted indoles by means of the reactions of indolylboronic acids with electrophilic compounds are considered the methods of choice for modifying indoles because indolylboronic acids are easily available, stable, non-toxic and new reactions using indolylboronic acids have been described in the literature. Thus, the aim of this review is to summarize the methods available for the preparation of indolylboronic acids as well as their chemical transformations. The review covers the period 2010–2019.

## 1. Introduction

**Figure 1:**
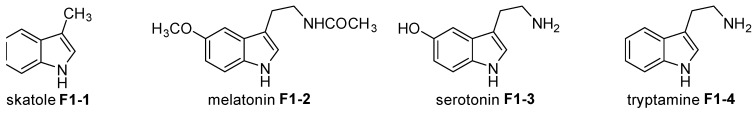
Structures of naturally occurring indole derivatives.

Indole is one of the simplest fused heterocyclic compounds known to act as a key scaffold of many naturally occurring molecules. For example, skatole (**F1-1**), melatonin (**F1-2**), serotonin (**F1-3**), and tryptamine (**F1-4**) are all well-known indole-based compounds (Figure 1). The functionalization of indoles, including the determination of their biological activities, has been the subject of considerable scientific research interest. Indeed, substituted indoles have been reported as affinity probes for the 5-hydroxytryptamine receptor [1], selective tumor-associated carbonic anhydrase inhibitors [2], carboxamide spleen tyrosine kinase inhibitors [3], selective inhibitors with broad hepatitis C virus genotype activity [4], Nav1.7 inhibitors [5], and TNFα inhibitors [6]. Further, the indole skeleton, as a constituent of naturally occurring substances, has been modified to furnish (−)-clavicipitic acid [7], breitfussin B [8], alstoscholarisines A and E [9], (+)-kopsihainanine A [10], and (−)-goniomitine [11].

**Scheme 1:**
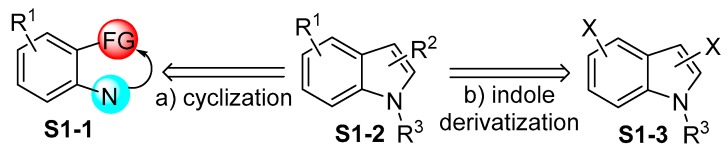
Principal approaches for the synthesis of substituted indoles.

The synthetic approaches for the preparation of substituted indoles can be divided into two main groups. The first group consists of methodologies involving both transition-metal-catalyzed and transition-metal-free cyclization strategies for *ortho*-substituted benzenes bearing the nitrogen-containing functional group, which are mostly substituted anilines (Scheme 1, route a). The cyclization pathway for the preparation of indoles is represented by the Baeyer-Emmerling [12], Bartoli [13], Fischer [14], Fukuyama [15], Gassman [16], and Larock [17] methods of indole synthesis. The second approach to indole synthesis is based on the modification of already functionalized indoles by means of traditional electrophilic [18,19] and nucleophilic [20] substitution reactions or transition-metal-catalyzed reactions of indolyl substrates (Scheme 1, route b) [21,22,23,24,25].

**Scheme 2:**
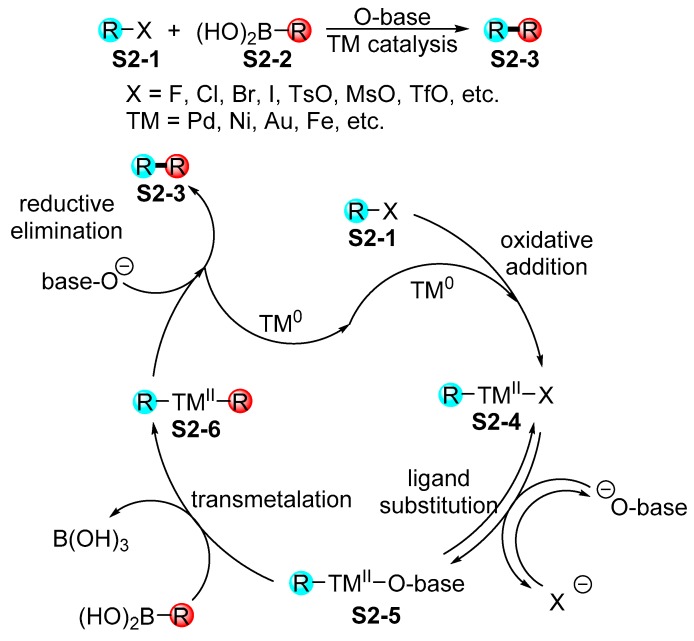
General scheme for the Suzuki-Miyaura reaction.

Transition metal-catalyzed cross-coupling reactions represent an indispensable tool for organic synthesis when considering the formation of C−C and C−heteroatom bonds. Among the available cross-coupling reactions, the Suzuki-Miyaura reaction [26,27] is the method of choice in terms of designing the construction of a C−C bond under mild reaction conditions. The Suzuki-Miyaura reaction involves a simple reaction between boronic acids **S2-2** or boronates/trifluoroborates and electrophilic templates **S2-1**, as represented by halides, tosylates, triflates, or phosphates, in the presence of a base and transition metal catalyst. The good availability, low toxicity, and high stability of boronic acids all explain the widespread popularity of the Suzuki-Miyaura reaction. The reaction mechanism behind the Suzuki-Miyaura reaction involves a complex process [28] featuring oxidative addition, ligand substitution, transmetalation, and reductive elimination as the main reaction steps (Scheme 2).

Two reaction pathways can be envisioned when designing the synthesis of substituted indoles by means of the Suzuki-Miyaura reaction. The first approach is based on the cross-coupling of aryl- or heteroarylboronic acids with electrophilic indolyl templates. Selected examples show how chloro- [29], bromo- [30], bromoindolyl phosphate [31], and triflates [32] are all capable of coupling with boronic acids under mild reaction conditions. Yet, a wide variety of allylic alcohols [33], heterocyclic halides [34], bromoenol phosphates [35], sulfones [36], and sulfonates [37] have been used as electrophilic templates in reactions with indolylboronic acids under Suzuki-Miyaura reaction conditions involving, for example EvanPhos [38] and tri-*tert*-butylphosphine ligand [39], nickel [40], and rhodium [41] catalysts, as well as asymmetric transformations [42,43]. The abovementioned examples illustrate the practical application of the Suzuki-Miyaura reaction by means of both electrophilic and nucleophilic indolyl templates for the synthesis of substituted indoles. Aside from the Suzuki-Miyaura reaction, indolylboronic acids constitute an important type of indole intermediate, which has many applications in the synthesis of functionalized indoles. The lack of a comprehensive English written review summarizing the basic reactivities of indolylboronic acids prompted us to focus on this topic.

## 2. Synthetic Approaches to Indolylboronic Acids

### 2.1. Introduction

**Scheme 3:**
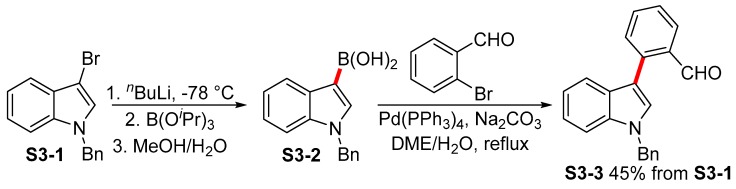
Bromine-lithium exchange reaction for the synthesis of 3-indolylboronic acid **S3-2**.

The traditional approach to indolylboronic acids is based on the halide-to-lithium exchange reaction by means of *^n^*BuLi. Selected examples reported by Liu [44] show the synthesis of 3-indolylboronic acid via the reaction of 3-bromoinodole **S3-1** with *^n^*BuLi followed by the addition of triisopropyl borate. The boronic acid **S3-2** that was formed in this way was used in the subsequent Suzuki-Miyaura reaction, affording the indole **S3-3** with an overall isolated yield of 45% (Scheme 3).

**Scheme 4:**
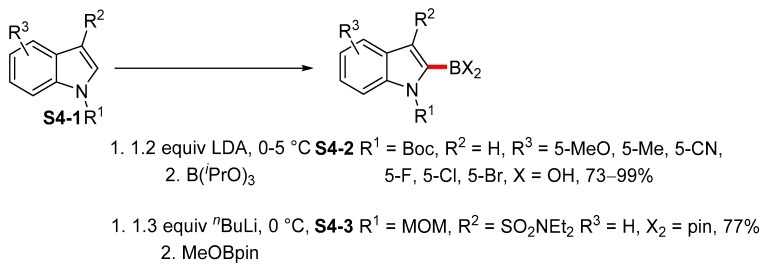
The synthesis of indolylboronic acids by means of *ortho*-metalation.

The alternative synthesis of indolylboronic acids by means of the reaction of lithiated indoles with borates takes advantage of *ortho*-metalation (Scheme 4). When compared to the bromine-lithium exchange reaction, ortho-lithiation reduces the number of steps required to synthesize indolylboronic acids; however, the presence of a directing group is required. Vazquez [45] and Snieckus [46] developed a protocol for indole lithiation by means of *^n^*BuLi or LDA. The isolated yields of indolylboronic acid **S4-2** and boronate **S4-3** were within the yield range of 77−99%. The regioselectivity of the published lithiation in these cases is driven by the presence of a directing group as well as by the highest pKa value of the H2 hydrogen [47].

**Scheme 5:**
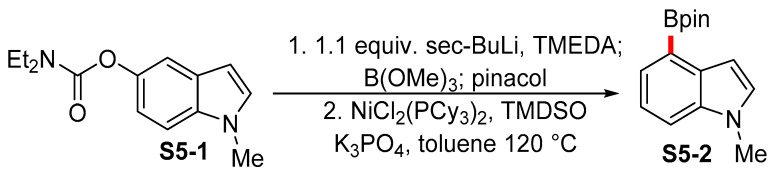
Carbamate as a traceless directing group for the *ortho*-lithiation of indoles.

A similar approach was reported by Garg in relation to the 4-indolylboronic acid pinacol ester **S5-2** (Scheme 5) [48]. The carbamate group was used as a traceless directing group, which allows for regioselective lithiation in position 4 of the indole scaffold. Once the Bpin fragment was introduced, the carbamate moiety was removed by means of a Ni-catalyzed reduction by 1,1,3,3-tetramethyl-disiloxane (TMDSO).

### 2.2. Miyaura Borylation en route to Indolylboronates

**Scheme 6:**
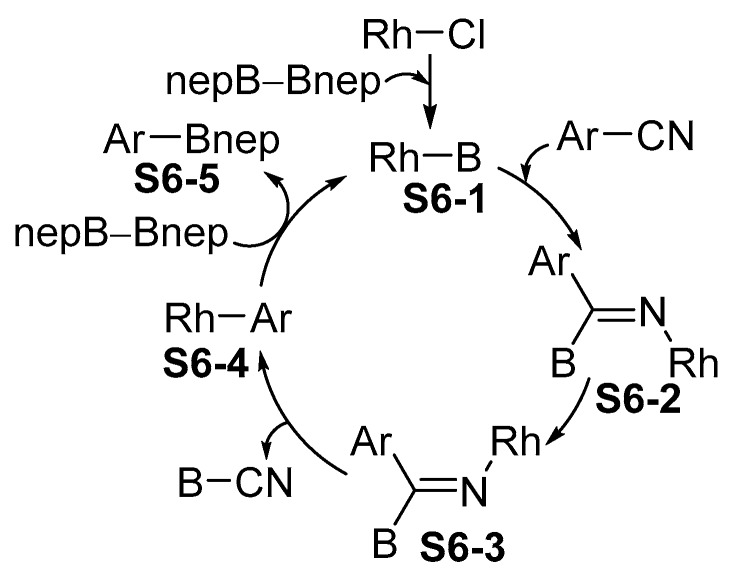
Proposed mechanism for the rhodium-catalyzed borylation of nitriles.

Miyaura borylation is a popular method for the synthesis of boronic acids and their derivatives. The typical borylation setup makes use of aryl/hetaryl halides, borylation reagents, and transition metal catalysts. Both iodide and chloride are able to couple with either pinB-Bdan [49] or diisopropylaminoborane [50] under palladium catalysis conditions (Table 1, entries 1 and 2). The isolated yields of indolylboronates ranged from 47−85%. The borylation was also performed in the presence of copper [51], cobalt catalysts [52], and copper-iron cooperative catalysis [53] (Table 1, entries 3−5). While Miyaura borylations of C−Cl, C−Br, and C−I bonds are quite common, the borylation of C−F bonds remains rare. This could be attributed to the relatively strong carbon−fluorine bond, which exhibits high dissociation energy and the shortest bond length in the organic halide series. Despite the unfavorable properties of C−F bonds in relation to transition-metal-catalyzed reactions, some borylations of fluoroindoles have been reported in the literature. Lee [54] and Ruben [55] utilized cobalt and nickel catalysts to carry out their indole borylations (Table 1, entries 6 and 7). Aside from traditional nucleophiles, aryl and hetaryl nitriles also react during borylative cross-coupling (Table 1, entry 8) [56,57]. The reaction is catalyzed by a rhodium catalyst, while bis(neopentyl glycolato)diboron (nepB-Bnep) is used as a borylation reagent. The rhodium precatalyst is activated by the reaction with nepB-Bnep. Then, the addition of activated catalyst **S6-1** to nitrile along with *E*/*Z* isomerization gives complex **S6-3**. This complex undergoes β-aryl elimination, thereby affording arylated rhodium species **S6-4**, which indicates that the synthesis of boronates **S6-5** is accomplished (Scheme 6). Alternatively, the borylation of haloindoles can be performed under transition-metal-free conditions. The reported procedures rely on either light-mediated borylation with pinB-Bpin under continuous-flow conditions (Table 1, entry 9) [58] or potassium-methoxide-mediated borylation with silylborane PhMe_2_Si-Bpin (Table 1, entry 10) [59,60] under mild reaction conditions. Transition-metal-free borylation has also been performed by mixing indolyldiazonium tetrafluoroborate with tetrahydroxydiboron; however, the yield of indolylboronic acid was only 35% [61].

**Scheme 7:**
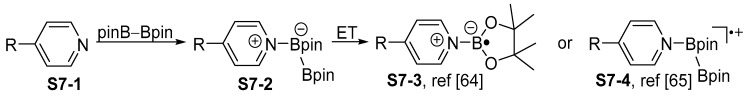
A mechanistic proposal for transition-metal-free borylation.

A decarboxylative cross-coupling represents an alternative procedure for the synthesis of indolylboronic acids. The borylation reaction can be performed by starting with either carboxylic acids [62] or thioester [63] under palladium or rhodium catalysis (Table 2, entries 1 and 2). The decarboxylative indole borylation has also been performed under transition-metal-free conditions (Table 2, entries 3 and 4). The reported protocols make use of *N*-hydroxyphthalimide esters, which are treated with pinB−Bpin under different conditions. Fu et al. [64] rely on the isonicotinate *tert*-butyl ester when refluxing in trifluorotoluene (TFT), while Glorius et al. [65] make use of pyridine along with a 400-nm light-emitting diode (LED). A common feature of both decarboxylative borylations is the activation of pinB−Bpin by means of pyridine **S7-1**, thereby affording the ate complex **S7-2** (Scheme 7). Next, the ate complex **S7-2** undergoes a transformation into radical intermediates via electron transfer, and the formed radicals **S7-3 [64]** and **S7-4 [65]** are combined with the activated *N*-hydroxyphthalimide ester to accomplish the borylation sequence. The isolated yields of indolylboronic acid pinacol esters are moderate in all cases.

### 2.3. Transition-Metal-Catalyzed Borylation of Indoles by Means of C–H Activation

**Scheme 8:**
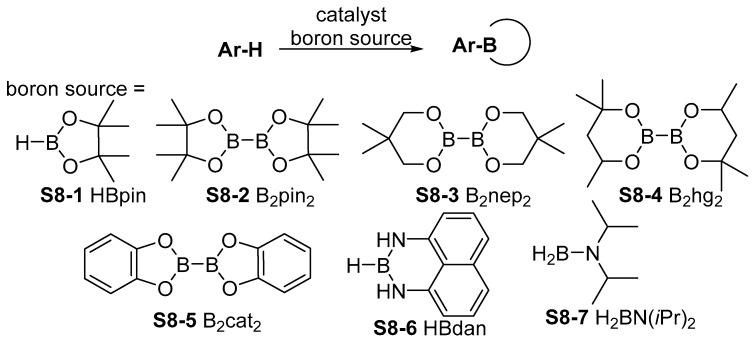
Common boron sources for the C−H borylation of arenes.

Since the discovery of the iridium-mediated C−H borylation reactions of aromatic substrates by Iverson and Smith [66], as well as the later improvement of the reaction conditions into iridium-catalyzed C−H borylation with B_2_pin_2_ or HBpin by Hartwig [67], the transition-metal-catalyzed borylation reactions have become one of the most versatile tools for the synthesis of borylated aromatic compounds. Heteroarenes, similar to indoles, can also undergo C−H borylation. The site selectivity of the reaction is an issue, as theoretically up to seven different sites can undergo C−H functionalization. Typically, in the case of unsubstituted indoles, the site selectivity is driven by electronic effects, as the reaction proceeds most readily at the C2 position where the C−H bond is the most acidic, and therefore, the most reactive. It is important to note, however, that selectivity can be overridden by steric factors. Several transition metals can be used for the borylation reactions of indoles, including iridium, platinum, nickel, and cobalt, although iridium-based catalytic systems with bipyridine ligands remain the most prevalent. In recent years, several site-selective and non-selective C−H borylation protocols of indoles have been developed, principally focusing on the recyclability of the expensive catalysts and site selectivity. Several boron sources can be used for the transition-metal-catalyzed C−H borylations of arenes (Scheme 8). The most common sources, namely HBpin (**S8-1**) and B_2_pin_2_ (**S8-2**), were established by Hartwig in relation to the transition-metal-catalyzed C2 borylation of indoles in the early 2000s [68].

#### 2.3.1. C2-Selective Borylation

**Scheme 9:**
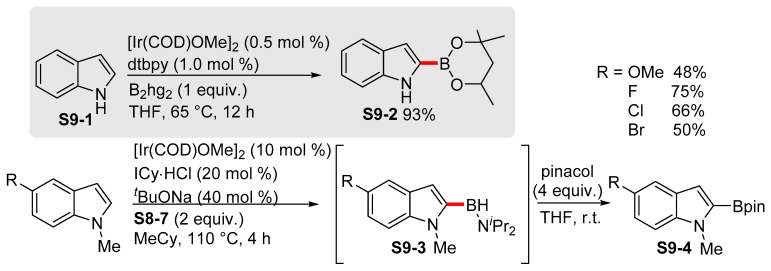
Iridium-catalyzed borylation of indoles using alternative boron sources to HBpin and B_2_pin_2_.

Recently Hartwig developed a protocol for the iridium-catalyzed borylation of arenes using B_2_hg_2_ (**S8-4**), which could also be successfully applied to the C2 borylation of indoles (**S9-1**) (Scheme 9) [69]. Tobisu and Chatani developed a one-pot protocol for the Ir/NHC carbene-catalyzed selective borylation of indoles using diisopropylamino borane (**S8-7**) as the boron source [70]. The formed B−N products **S9-3** could not be isolated, although they could be turned into pinacol indol-2-ylboronates **S9-4** with good isolated yields (Scheme 9).

**Scheme 10:**
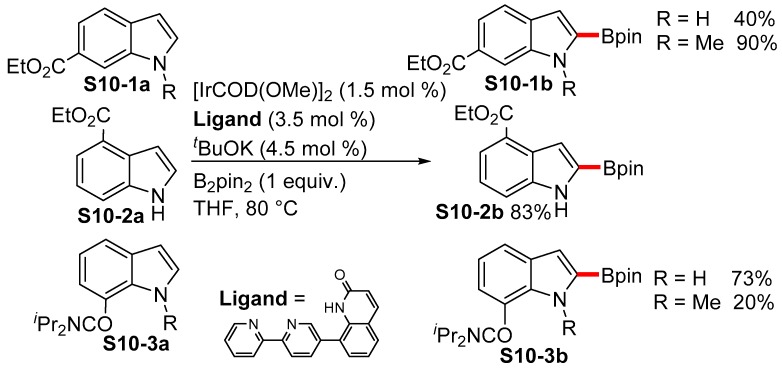
Selective C2 borylation utilizing L-shaped ligands.

Chattopadhyay recently developed L-shaped ligands for the selective borylation of the remote sites (*meta* and *para*) of aromatic esters [71] and amides [72], and the protocol could also be applied to indoles. Indoles that lacked the ester- or amide-directing group did not undergo the borylation reaction, most likely due to the preferential complexation of the indole NH group to the catalyst, thus inhibiting the borylation. Interestingly, following the introduction of the directing group onto the indole scaffold, the borylation proceeded without *meta-* or *para-*selectivity, but always into the C2 position (**S10-1b,2b,3b**), which suggests that electronic effects in the case of indoles **S10-1a,2a,3a** played a crucial role in the site selectivity (Scheme 10). Exchanging the L-shaped ligands for simple tbdpy ligands under these reaction conditions resulted in the formation of complex mixtures of unselectively borylated indoles.

**Scheme 11:**
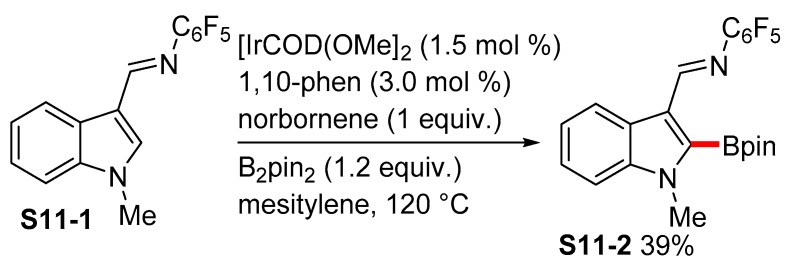
Aldimine-directed borylation of the protected indolyl aldehyde **S11-1**.

As shown above, the ester and amide functional groups remain stable during the iridium-catalyzed C−H borylation, although the aldehyde group is not stable. Therefore, Ito and Ishiyama developed a protocol whereby heteroaromatic pentafluorophenyl aldimines undergo iridium-catalyzed C−H borylation in moderate to high yields, with one example being the indole substrate **S11-1** [73]. The aldimine functional group serves as both a directing group and a protection group, and it can be easily hydrolyzed so as to produce aldehydes (Scheme 11).

**Scheme 12:**
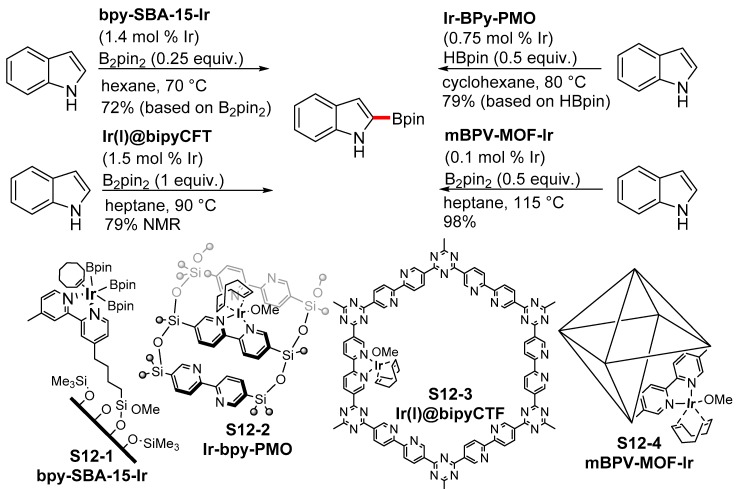
Selective C2 borylation of indole using heterogeneous catalysts, including the represented structures of the catalysts.

In an effort to enhance the prospects of catalyst recovery and recycling, several groups have developed iridium-based heterogeneous catalysts for the C−H borylation of aromatic and heteroaromatic compounds. For instance, Jones et al. prepared and applied the mesoporous silica-supported bipyridine-based iridium catalyst bpy-SBA-15-Ir (**S12-1**) for the borylation of indole (Scheme 12) [74]. The catalyst did not reach a turnover number (TON) higher than 136, although it could be repeatedly reused following simple filtration with only the gradual loss of activity with subsequent runs. Similarly, Inagaki developed the heterogeneous catalyst Ir-bpy-PMO (**S12-2**), which in combination with the inexpensive boron source HBpin facilitated the C−H borylation of aromatic and heteroaromatic compounds, while the catalytic activity of Ir-bpy-PMO was comparable to that of homogenous catalytic systems (Scheme 12) [75]. Another example of a heterogeneous catalyst for C−H borylation reactions is based on the covalent triazine framework (CTF) prepared by Van Der Voort et al. They developed a bipyridine-based CTF and then metalated it with the Ir(I) complex so as to prepare an air-stable catalyst (**S12-3**) for the C−H borylation of various aromatic compounds, including indole [76]. The borylation reactions proceeded in non-polar solvent under mild reaction conditions, and the catalyst could be recycled at least five times with only a small decrease in activity (Scheme 12). Excellent results have been achieved with heterogeneous catalysis when using the bipyridine-based metal-organic framework (mBPV-MOF-Ir) (**S12-4**) and the 1,10-phenanthroline-based metal-organic framework (mPT-MOF-Ir) catalysts developed by Lin et al. [77]. They used them for the C−H borylation of various aromatic and heteroaromatic substrates with very low catalyst loadings and excellent isolated yields of borylated products. Although prolonged reaction periods and high temperatures were required to achieve full conversion, the products were obtained by removing the catalyst via simple centrifugation and the catalysts could be recycled at least 15 times with no apparent loss of activity (Scheme 12). When using the mBPV-MOF-Ir catalyst, a TON of 9000 was achieved for the borylation of indole and the leached iridium content was found to be 0.3 ppm.

**Scheme 13:**
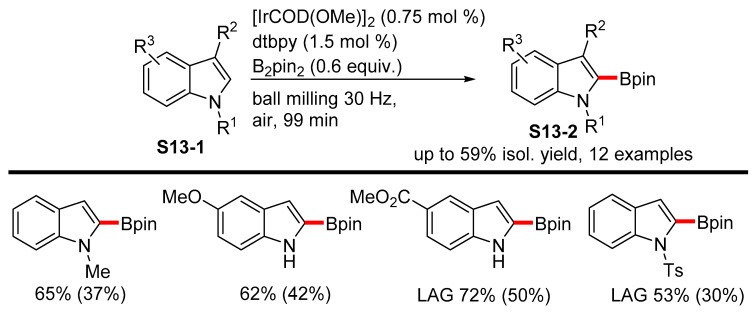
Mechanochemistry as applied for the iridium-catalyzed borylation of indoles.

Very recently, the first-of-its-kind mechanochemical iridium-catalyzed C−H borylation of indoles **S13-1** was described by Kubota and Ito. The borylation reaction took place in a steel jar in an air atmosphere while the starting compounds were ground by a steel ball for 99 min [78]. The authors report that the careful selection of the grinding apparatus was vital if the reaction was to give results. For substrates with melting points higher than 70 °C, liquid-assisted grinding (LAG) was needed, although a catalytic amount of the organic solvent was sufficient. Several variously substituted 2-indolyl boronates **S13-2** were prepared in this way. The isolated yields (in parentheses) differed substantially from the NMR yields of the products due to the reported fast hydrolysis on silica gel (Scheme 13).

In an effort to render transition-metal-catalyzed borylation more economically feasible, several methods based on more abundant metals, such as cobalt, nickel, and platinum, have been developed in recent years. Some of the developed protocols are also applicable for indoles, although in most cases, only a very narrow indole substrate scope was tested. In the identified publications, only indoles with unsubstituted C2 positions were tested; therefore, the borylation proceeded selectively into the C2 position in all cases.

**Scheme 14:**
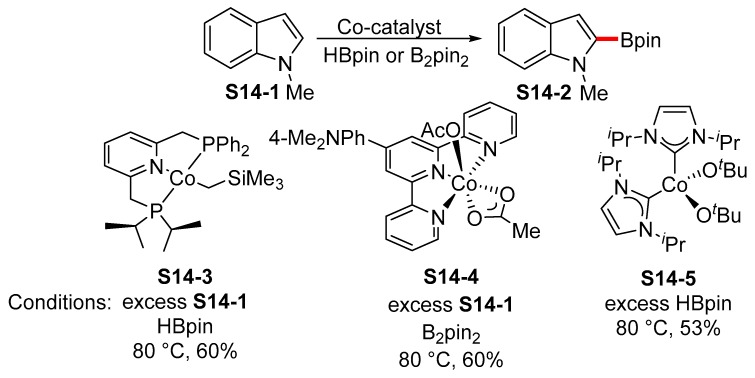
Cobalt-catalyzed borylations of the indole **S14-1**.

Chirik used a series of pincer-type ligand cobalt complexes for the development of the cobalt-catalyzed borylation of aromatic compounds [79]. The complex **S14-3** proved to be effective in terms of the selective borylation of indole **S14-1** into the C2 position (Scheme 14). Later, Chirik broadened his application of the cobalt complexes for C−H borylation by means of the preparation and evaluation of a new, air-stable and easily preparable cobalt precatalyst, namely (^Ar^Tpy)Co(OAc)_2_ (**S14-4**), which was also applicable for the borylation of indoles (Scheme 14) [80]. Recently, Smith and Maleczka have shown that a cobalt-based catalyst supported by NHC ligands (**S14-5**) can also be used for the borylation of benzylic and heteroaromatic compounds, including the indole **S14-1**, in moderate yields (Scheme 14) [81].

**Scheme 15:**
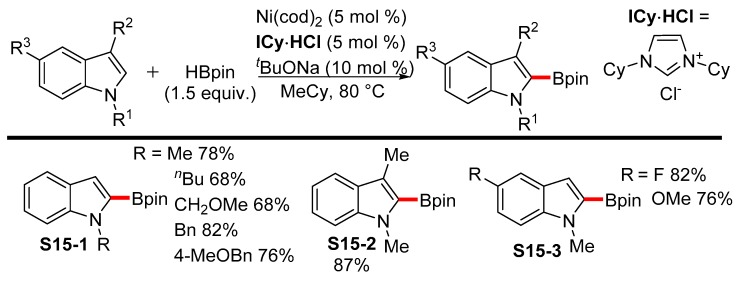
Nickel-catalyzed borylation of indoles.

**Scheme 16:**
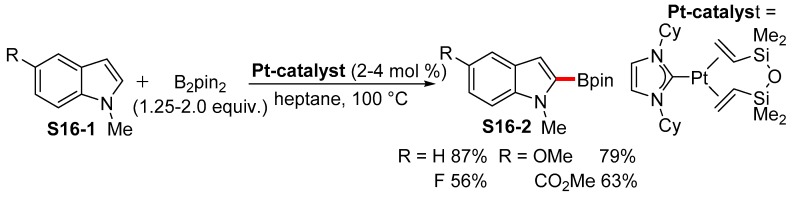
Platinum-catalyzed borylation of indoles developed by the Chatani group.

Nickel-catalyzed C2 borylations of various arenes and heteroarenes have been developed by the Chatani group [82]. In this instance, the catalytic system consisted of the transition metal and an NHC ligand. The reaction proceeded selectively into the C2 position in the case of indoles with nitrogen atoms variously substituted by the alkyl or benzyl groups (**S15-1**). Similar selectivity was achieved when positions 3 and 5 were substituted by the methyl (**S15-2**) or halogen and methoxy groups (**S15-3**), respectively (Scheme 15). The authors managed to employ cheap and air-stable Ni(OAc)_2_ rather than Ni(cod)_2_ for the gram-scale preparations, and they achieved excellent results. Similar conditions were used for the borylation of indoles under platinum catalysis conditions, although HBpin proved to be unreactive; therefore, B_2_pin_2_ was used as the boron source [83]. The reaction with B_2_pin_2_ proceeded selectively at the 2 position, and it offered the indoles **S16-2** in moderate to very good yields (Scheme 16). Iwasawa also applied platinum catalysis for the borylation of indoles using the PSiN-pincer platinum complex, albeit with diminished selectivity [84].

#### 2.3.2. C3-Selective Borylation

**Scheme 17:**
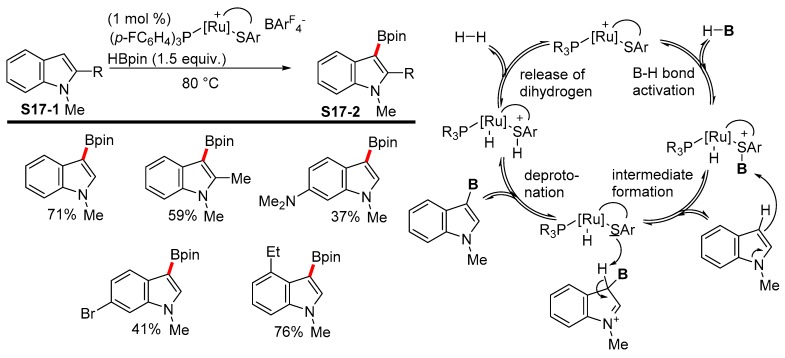
Ruthenium-catalyzed C3 borylation with HBpin, including the outlined mechanism.

In 2013, Oestreich reported the new and synthetically useful C3-selective borylation of indoles **S17-1** by means of the catalytic generation of borenium ions from HBpin [85]. The protocol required no added base, as H_2_ was released as the sole by-product and allowed to install the Bpin group onto the indole substrate via a S_E_Ar reaction mechanism. Over the course of the reaction, the B−H bond is split by the ruthenium(II) thiolate complex, thereby affording ruthenium(II) hydride and borenium ions, which undergo an attack by the most nucleophilic site of the indole substrate. The subsequent deprotonation offers C3-borylated indoles in moderate to high yields (Scheme 17).

**Scheme 18:**
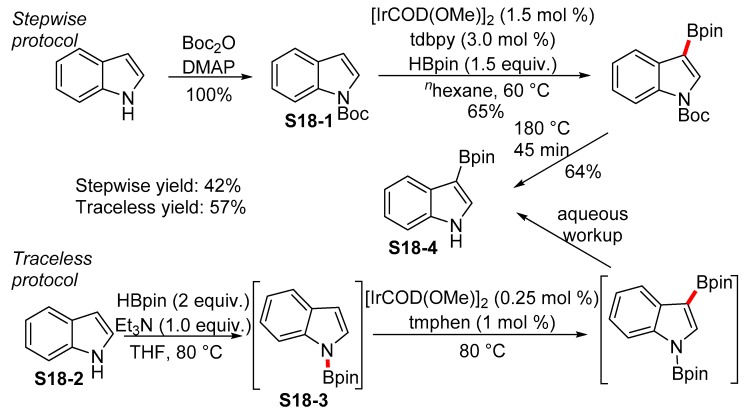
Stepwise and traceless protecting group protocols that complement C3 borylations of indoles.

The regioselectivity of the iridium-catalyzed C−H borylation of indoles can be altered by installing a bulky group onto the indole substrate. The *tert*-butoxycarbonyl (Boc) group can be used in this way for nitrogen-containing heterocycles such as pyrroles, indoles, azaindoles, and pyrazoles where the *N*-Boc-protected pyrroles and indole **S18-1** are selectively borylated into the 3 position [86]. However, the installation and removal of the directing group extend the time required for the overall procedure. Therefore, it is desirable to use a directing group that can be installed and removed without requiring the isolation of the intermediates. The protocol developed by Smith and Maleczka can be used as an example here, with the Bpin group serving as a traceless directing group for the selective C−H borylation of indoles into the 3 position (Scheme 18) [87]. The indole **S18-2** is smoothly converted into the *N*-borylated indole **S18-3** by means of the reaction with HBpin. The addition of Et_3_N is essential if the reaction is to proceed, as the coordination of the lone nitrogen pair with the boron center in the HBpin renders the B−H bond hydridic enough to react with as weak an acid as **S18-2**. The addition of the iridium catalyst into the reaction mixture causes the C−H borylation to proceed in the C3 position, while the aqueous workup gives 3-borylated indole **S18-4** in a 57% yield (Scheme 18).

**Scheme 19:**
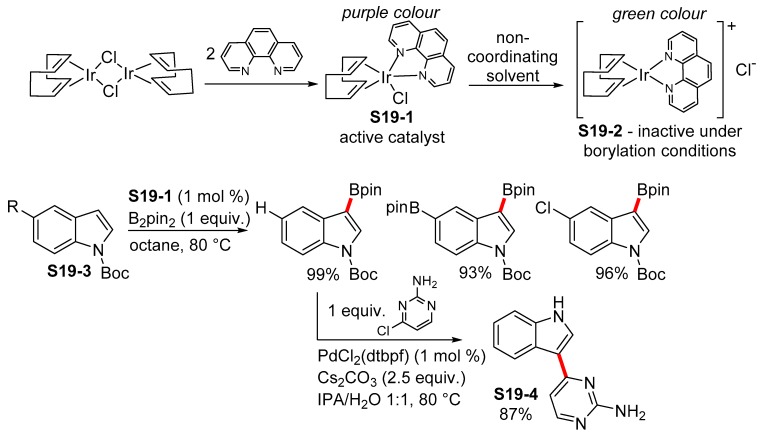
C3-selective borylation of Boc-protected indoles based on the cheap 1,10-phenanthroline ligand.

Colacot et al. studied the causes of prior inconsistent results and diminished the catalytic activity of the Ir complexes formed in situ using the simple and cheap ligand 1,10-phenanthroline. They determined that catalytically inactive cationic [Ir(COD)(phen)]^+^Cl^-^ (**S19-2**) is rapidly formed in non-coordinating solvents, thereby diminishing the reactivity (Scheme 19). The authors managed to isolate the active complex [Ir(Cl)(COD)(phen)] (**S19-1**) and to utilize it for the selective borylation of the Boc-protected indoles **S19-3**. They achieved excellent results and then applied the catalyst based on the cheap ligand for the one-pot total synthesis of meridianin G (**S19-4**), thereby achieving an excellent overall yield [88].

#### 2.3.3. C6-, C7-Selective Borylation

**Scheme 20:**
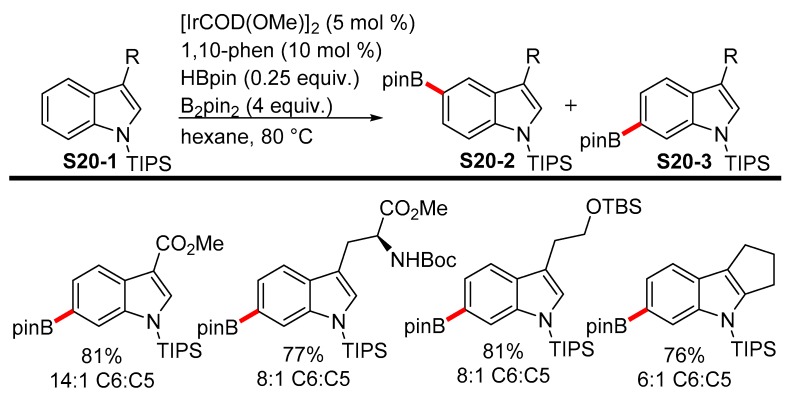
C6-preferential iridium-catalyzed borylation of 3-substituted indoles.

Only one example of the selective C6-borylation of indoles can be found in the literature, and even it is not strictly C6-selective but rather C6-preferential. It was developed by Baran et al. in an attempt to achieve the first total syntheses of verruculogen and fumitremorgin A. The C6 selectivity is achieved by means of the careful substrate selection of only 3-substituted indoles **S20-1** and the protection of the indole nitrogen by the robust TIPS group, thereby disabling the C−H activation reaction in the otherwise more reactive positions C2, C3, or C7 [89]. By optimizing the reaction conditions, the authors achieved good isolated yields of **S20-3** as well as up to 14:1 C6:C5 selectivity on a limited substrate scope (Scheme 20).

**Scheme 21:**
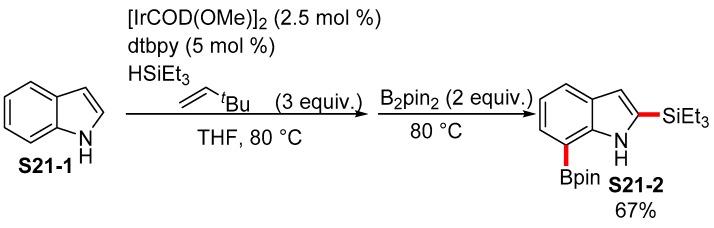
Ir-catalyzed silylation/borylation sequence for the selective borylation of the C7 position.

Selective transition-metal-catalyzed C−H functionalization at the 7-position typically requires a substituent to be present at the 2-position so as to block reactivity at that site [90,91,92]. An example from the recent literature was developed by Takai, who exploited the iridium-catalyzed silylation of indole (**S21-1**) to functionalize the 2-position, followed by iridium-catalyzed borylation in the 7-position in a one-pot manner so as to prepare **S21-2** (Scheme 21). 

**Scheme 22:**
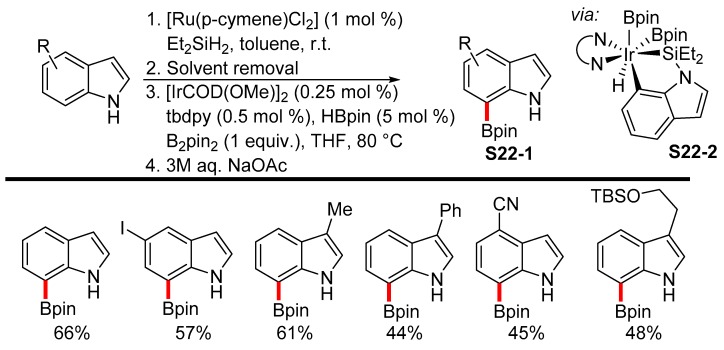
One-pot C7-selective borylation of indoles and selected examples.

The requirement for an occupied 2-position can be avoided by means of the introduction of a silyl-directing group onto the nitrogen atom, as demonstrated by Hartwig in his one-pot protocol consisting of the Ru-catalyzed silylation of indoles, followed by the Ir-catalyzed borylation of the formed silylindole. The mild hydrolysis of the silyl group then selectively afforded the C7-borylated indoles **S22-1** (Scheme 22) [93]. The borylation selectivity most likely arises from the formation of the five-membered metallacycle **S22-2** through the C−H bond cleavage at the 7-position rather than of the four-membered metallacycle at the 2-position. Sperry applied an analogous approach for the synthesis of indolequinone natural products where even 5- and 6-methoxy-substituted indoles reacted selectively at the 7-position, thereby broadening the applicability of the developed protocol [94].

**Scheme 23:**
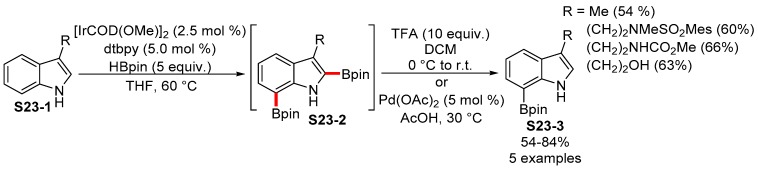
Selective C7 borylation using the diboronation/protodeboronation sequence developed by Movassaghi.

Inspired by Hartwig’s protocol, Movassaghi et al. developed a more streamlined C7-selective borylation of 3-alkylindoles. Their design was based on the diboronation/protodeboronation sequence. The authors managed to develop reaction conditions wherein the 3-substituted indoles **S23-1** underwent iridium-catalyzed C2/C7 diboronation with an excess of HBpin so as to form **S23-2** *in situ*, and subsequently, selective C2 protodeboronation either with a large excess of trifluoroacetic acid or a catalytic amount of Pd(OAc)_2_ in AcOH (Scheme 23) [95]. Smith and Maleczka later expanded upon this protocol. They developed a complementary method for selective C2 deborylation using the iridium-catalyzed deborylation of 2,7-diborylated indoles to prepare 7-borylated indoles [96]. They also determined that bismuth(III) acetate can be used for the C2 and C7 protodeboronation of indoles. They prepared a series of diborylated and triborylated indoles that underwent selective C2 and C7 deborylation with Bi(OAc)_3_ in methanol so as to prepare mono- and bisborylated indoles [97]. Recently, the diboronation/protodeboronation method was used by Burns et al. to achieve the total synthesis of Ts-discorhabdin E from tryptamine [98].

#### 2.3.4. Non-Selective Borylations

**Scheme 24:**
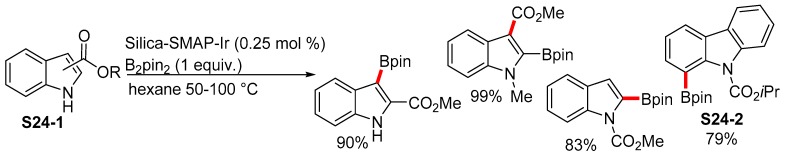
Ester-directed iridium-catalyzed borylation of indoles.

Several non-selective methods for the borylation of indoles have emerged over the last decade, which either produce multi-borylated indoles or else are highly substrate-specific and differently substituted indoles undergo borylation at different sites [99]. One such method concerns the ester-directed borylation of sulfur-, oxygen-, or nitrogen-containing five-membered heterocycles developed by Sawamura, whereby indoles **S24-1** bear the ester functional group borylate at either the 2- or the 3-position depending on where the ester group resides. If the ester group is present at the nitrogen atom, then the borylation occurs at the 2-position, and it only occurs at the 7-position (**S24-2**) when the 2- and 3-positions are occupied (Scheme 24) [100].

**Scheme 25:**
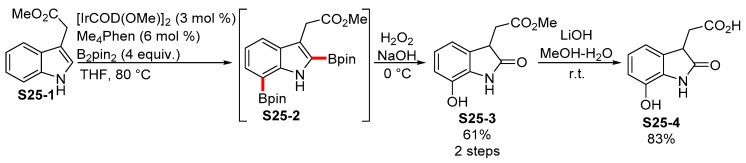
The short total synthesis of 7-hydroxyoxindole-3-acetic acid utilizing a selective diborylation/oxidation sequence.

As mentioned above, the borylations of indoles often proceed at multiple sites, most frequently at the C2 and C7 positions [101]. Protodeboronation at C2 can lead to selective C7 borylation, although that is not the only application of the inherent reactivity regarding indole borylations. Sperry applied a diborylation/oxidation protocol for the short total synthesis of an endogenous plant metabolite 7-hydroxyoxindole-3-acetic acid **S25-4**, which required only one chromatography separation (Scheme 25) [102]. Utilizing the Me_4_Phen ligand and an excess of B_2_pin_2_ was crucial for achieving the full conversion of the **S25-1** into **S25-2**.

**Scheme 26:**
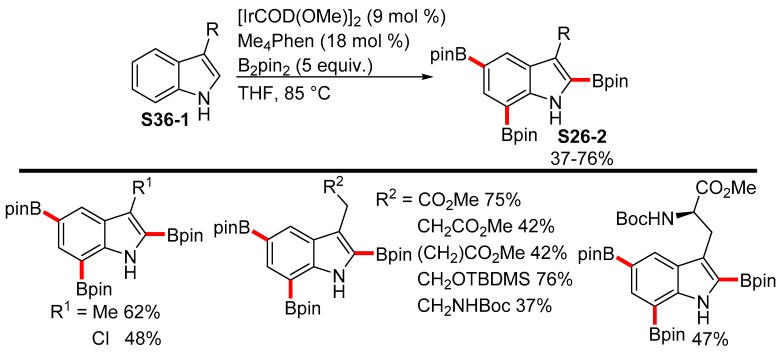
2,5,7-Selective threefold borylation of 3-substituted indoles.

Sperry also developed a reaction condition for the threefold borylation of 3-substituted indoles **S26-1** by employing iridium catalysis with the Me_4_Phen ligand. During the course of the reaction, the C−H borylation first proceeded quickly at the C2 and C7 positions, and then further, at the C5 position due to steric effects [103]. Several triborylated 3-substituted indoles **S26-2** were prepared by following the described protocol (Scheme 26).

**Scheme 27:**
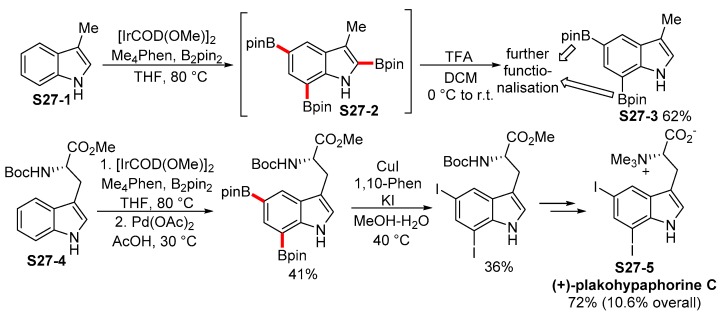
Triborylation/protodeboronation sequence for the synthesis of 5,7-functionalized indoles and the total synthesis of (+)-plakohypaphorine C.

Sperry then expanded the protocol to achieve the synthesis of 5,7-diborylated-3-methylindole **S27-3** from 3-methylindole **S27-1** utilizing the triborylation/C2-protodeboronation sequence in a one-pot manner (Scheme 27) [104]. The author showed that the two boryl groups in positions 5 and 7 of the **S27-3** can be further functionalized in a selective or non-selective way so as to prepare valuable indoles bearing the aryl group, methoxy group, or halogen atoms in the aforementioned positions. Additionally, this method was applied to achieve the total synthesis of (+)-plakohypaphorine C (**S27-5**) from Boc-protected L-tryptophan methyl ester (**S27-4**) (Scheme 27).

### 2.4. Cyclization Protocols for the Synthesis of Indolylboronic Acid Derivatives

Thus far, the synthesis of indolylboronic acids or esters has been achieved via the manipulation of the indolyl moiety. The opposite approach, which consists of the cyclization of the substituted anilines, can also be used to synthesize 3-indolylboronic acids or boronates (Table 3). The cyclization strategy can be performed under transition-metal-free conditions by means of a stoichiometric amount of BCl_3_ [105] or a catalytic amount of B(C_6_F_5_)_3_ [106] (Table 3, entries 1 and 2). Palladium [107] and gold [108] catalysts can also be used for the same cyclization, affording 3-indolylboronates in comparable isolated yields. The common feature of the above-mentioned protocols is the ability to provide rapid access to 2-subtituted-3-indolylboronates in good isolated yields and wide substrate scopes. Based on both experimental evidence and theoretical investigation [109], plausible mechanisms have been proposed to explain borylative cyclizations. Generally, simplified versions of the proposed mechanisms rely on the activation of the triple bond by means of Lewis acid complexation, and either transition metals or boron-based reagents can be used as acids (Table 3, entry 5). This complexation facilitates the addition of nitrogen to the triple bond. The synthesis of regioisomeric 2-indolylboronates is also feasible [110], although a procedure starting from 2-iodoanilines and MIDAB-acetylene under typical Miyaura borylation conditions would have to be used.

**Scheme 28:**
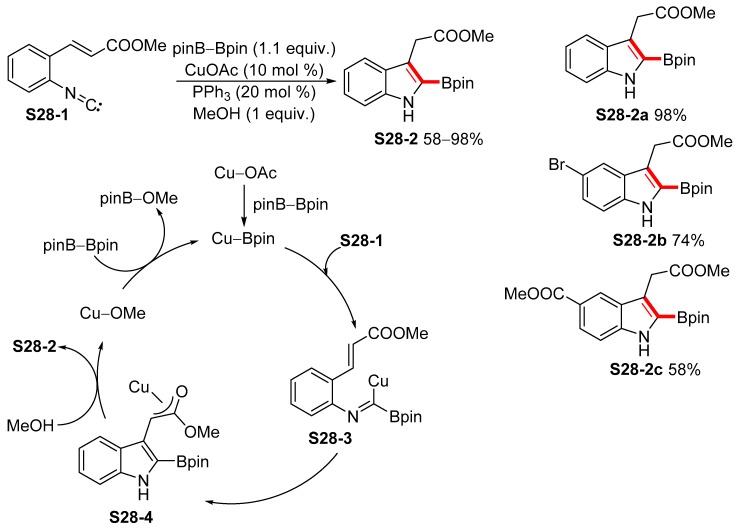
Borylative cyclization of *ortho*-substituted phenylisocyanides **S28-1** catalyzed by copper(I) acetate.

Save for the *ortho*-substituted aniline, the *ortho*-substituted phenylisocyanides undergo smooth cyclization so as to give substituted 2-indolylboronic acid pinacol esters **S28-2** (Scheme 28) [111]. This methodology leads to excellent yields of the substituted indoles **S28-2**, although the presence of alkenyl species bearing an ester group is required. This requirement can be explained by a plausible mechanism. It is expected that the copper catalyst activates the pinB-Bpin, followed by a reaction with the isocyanide **S28-1**. The formed complex **S28-3** then undergoes intramolecular addition so as to give copper enolate **S28-4** while the ligand substitution releases the 2-indolylboronates **S28-2**.

### 2.5. Lewis-acid-catalyzed Borylation of Indoles

**Scheme 29:**
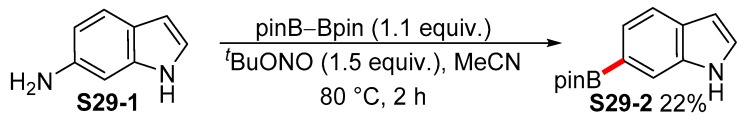
Aryldiazonium salts as the precursor of the synthesis of pinacolboronates.

It is clear that the transition-metal-catalyzed borylations of indoles have become the methods of choice when designing the functionalization of indoles via indolylboronic acid intermediates. Yet, transition-metal-free borylations offer a relatively simple and cheap route to indolylboronates under mild reaction conditions. In 2013 [112] and 2014 [113], Wang et al. described a procedure that allowed for the conversion of arylamines into indolylboronic acid pinacol esters by means of the reaction with pinB-Bpin in the presence of *^t^*BuONO (Scheme 29). The developed methodology was found to be applicable in relation to diverse aromatic and heteroaromatic substrates substituted with both electron-donating and electron-withdrawing groups. Overall, the isolated yields were within the range of 14−80%, and a single example of indolylboronate **S9-2** was isolated in a 22% yield. The authors proposed a single-electron transfer (SET) mechanism for their reaction [112]. 

**Scheme 30:**
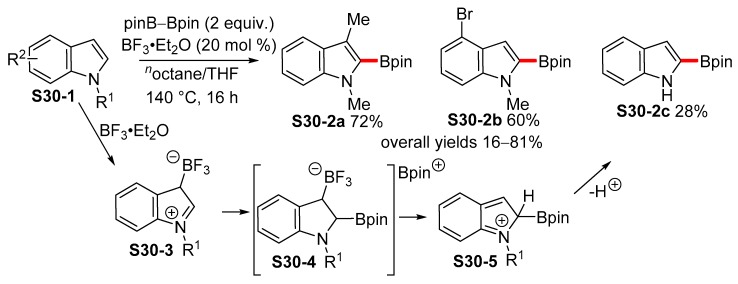
Lewis-acid-mediated borylation of indoles by means of pinB-Bpin and BF_3_∙Et_2_O.

A different approach comparable to the transition-metal-catalyzed borylation of indoles was reported by Zhang (Scheme 30) [114]. He observed that a catalytic amount of boron trifluoride diethyl etherate was able to catalyze the conversion of substituted indoles into indolylboronic acid pinacol esters. A wide range of indolylboronates were prepared by heating the starting compounds in *^n^*octane/THF to 140 °C. The author was able to synthesize indoles with sensitive bromine function **S30-2b**, unprotected indole **S30-2c**, as well as 2,3-disubstituted indole **S30-2a**. It is expected that the reaction proceeds through an initial attack of BF_3_ on the indole-affording intermediate **S30-3**. The intermediate **S30-3** then reacts with the pinB−Bpin to generate the dihydroindole **S30-4**. This intermediate **S30-4** is disproportionated to the carbocation **S30-5** and pinB−BF_3_^−^, which deprotonates **S30-5** so as to yield the indolylboronates **S30-2a-c**.

The example presented in Scheme 30 illustrates an application of formal electrophilic aromatic substitution as a tool for the borylation of electron-rich arenes. This concept was further exploited by Zhang [115], Oestreich [116], and Erker [117] (Table 4). 

Zhang made use of catecholborane (catBH) along with pentafluoroborane as the Lewis acid in the catalytic setup. The reaction proceeds at room temperature as disproportionation affording a mixture of the indolylboronic acid catechol ester and the corresponding 2,3-dihydroindoles in good isolated yields (Table 4, entry 1). This approach allows for easy access to a variety of borylated indoles. However, the convergent disproportionation reaction is able to increase the yield of borylated indoles up to a 98% yield. In this case, the borylation is performed at 120 °C. Under the above-mentioned conditions, the catalyst enables the conversion of indolines into indoles, thereby allowing for a shift in the equilibrium of disproportionation reactions. A similar approach was reported by Oestreich et al. (Table 4, entry 2) [116]. The same reaction conditions were applied, although the scope of the protocol was extended to electron-rich arenes. Interestingly, the reaction was accelerated when performed in the presence of norbornene or norbornadiene. Substantial progress was achieved by running the borylation reaction in the presence of bisborane **T4-3**, which allowed the borylation to proceed at room temperature with a good isolated yield of the corresponding boronates (Table 4, entry 3) [117].

**Scheme 31:**
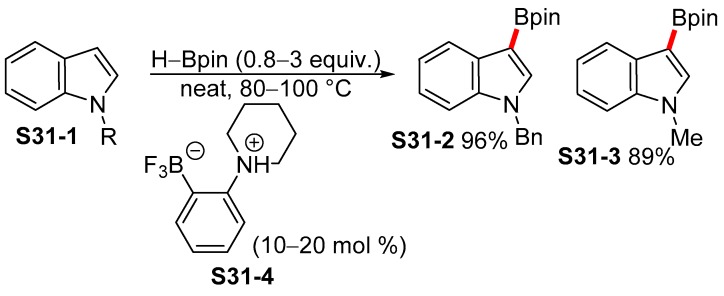
Frustrated Lewis pair C−H bond activation for the synthesis of indolylboronic acid pinacol esters.

In a series of papers, Fontaine showed that the frustrated Lewis pair **S31-4** can catalyze transition-metal-free C−H borylation (Scheme 31) [118,119,120,121]. The developed conditions are mostly applicable to five-membered heterocycles, thiophene, pyrrole, or furan. However, the substituted indoles **S31-2** and **S31-3** were prepared in excellent isolated yields.

**Scheme 32:**
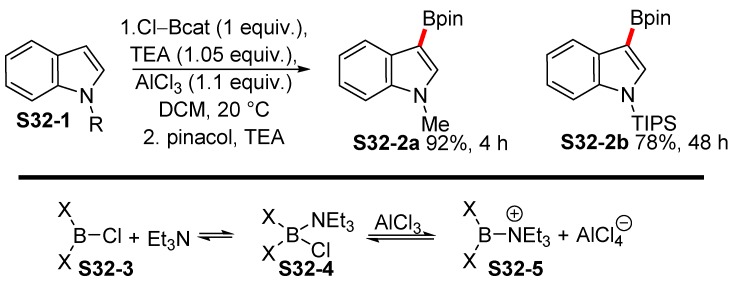
Borylation of arenes and heteroarenes with borenium cations.

A different type of electrophilic borylation was reported by Ingleson [122,123,124,125]. This reaction is applicable for the borylation of a wide range of heteroarenes and arenes, including pyrene and anthracene. The reaction can be performed by means of BCl_3_ [122] or the less reactive ClBcat [123,124] derivative. It was proposed that during the first step, the borylation reagent **S32-3** is activated by the presence of triethylamine and aluminum chloride, thereby affording the borenium cation **S32-5** as the main reactive species (Scheme 32). Then, the formed borenium cation reacts with the arenes, and following esterification (e.g., with pinacol), provides the products of borylation. Thus, both pinacol esters [122−124] and MIDA-boronates [122] are available via this protocol. 

**Scheme 33:**
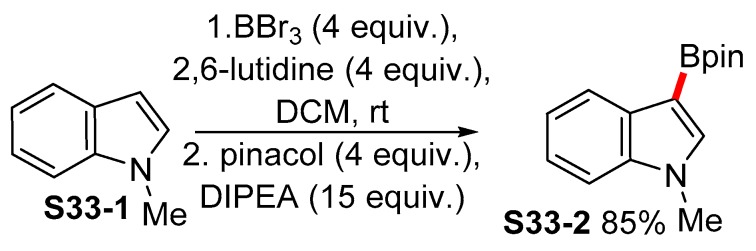
The synthesis of indolylboronic acid pinacol ester **S33-2** by means of boron tribromide-2,6-lutidine electrophilic borylation.

Recently, another different type of borylation reagent has been reported [126]. The protocol involves the treatment of electron-rich alkenes with four equivalents of boron tribromide in the presence of 2,6-lutidine at room temperature. The reaction is limited to electron-rich alkenes, and only one example of indole borylation is provided (Scheme 33).

The final option for the preparation of indolylboronic acids or boronates is based on the chemical manipulation of indoles bearing the −B(OH)_2_ or −B(OR)_2_ function. In this regard, regioselective Friedel−Crafts alkylation [127], boronate ligand interconversion [128], palladium or rhodium C−H activation [129,130], metal-free arylation [131,132], and other approaches [133] have emerged in the literature. Although these procedures allow for access to a novel type of indolylboronic acid or ester, they are not discussed in detail in the present paper because indolylboronic acid esters are already used as starting compounds.

## 3. Chemical Transformations of Indolylboronic Acid Derivatives

### 3.1. Formal Substitution of Boron Moieties by Transition-Metal-Catalyzed or Transition-Metal-Free Reactions 

Indolylboronic acids and their derivatives are all examples of simple and stable heteroaromatic compounds, which have found many applications in the field of organic synthesis, along with other aromatic and heteroaromatic boronates and boronic acids. Presumably, the simplest modification is represented by the protodeboronation that was reported by Macgregor and Lee (Table 5, entry 1) [134]. They found that 5 mol % of a gold catalyst facilitates the protodeboronation of boronic acids in the presence of dimethyl carbonate and water when using a microwave setup. As the reaction is carried out under non-acidic conditions, acid-sensitive functional groups (e.g., the THP group) are tolerated. The use of THF along with D_2_O allows for a simple deuterodeboronation. Based on density functional theory calculations, a mechanism explaining the observed reactivity has been proposed. A copper catalyst was used for the direct conversion of boronates, boronic acids, and organotrifluoroborates into the azides (Table 5, entry 2) [135]. 

The developed conditions saw the azidation performed in excellent isolated yields, while the coupling efficiency did not correlate with the electron-donating ability of the substituents. However, the isolated yield of azidoindole was only 51%. The reaction course could be followed colorimetrically as the dark brown solution turned light green upon completion. Recently, the rapid palladium-catalyzed [^11^C] C-cyanation of aryl- or heteroarylboronates was reported [136]. The reaction conditions allowed for the rapid conversion of the starting boronates by means of treatment with a [^11^C] NH_4_CN/NH_3_ system in the presence of a PdCl_2_(PPh_3_)_2_ catalyst. The high efficiency of the developed protocol was demonstrated via the cyanation of indolylboronate in a 90% radiochemical yield (rcy) and the synthesis of [^11^C] C-labeled cetrozole.

The *ipso*-substitution of B-moiety via a halogen atom represents an alternative approach for the synthesis of halogenated arenes. This transformation can be performed under transition-metal-free conditions by means of iodine and KF in dioxane at 80 °C (Table 6, entry 1) [137]. The isolated yield of iodoindole is high. However, this reaction is less important from a practical point of view, as indoles can be regioselectively lithiated at the 3-position by means of *^t^*BuLi [138]. 

The fluorination of indoles is more import, as fluorinated indoles are of interest due to their potential use in the field of medicine. Thus, Hartwig developed the copper(I)-catalyzed fluorination of pinacol boronic ester via an electrophilic fluorine source in THF at 50 °C (Table 6, entry 2) [139]. Overall, the isolated yields of the fluorinated arenes ranged from 29% to 77%, with a 44% isolated yield of 5-fluoroindole being achieved. Radiofluorination is a more attractive concept in relation to the fluorination of indoles. Two different procedures have thus far been reported (Table 6, entries 3 and 4). Both published procedures rely on Cu-mediated radiofluorination with different fluoride sources. Krasikova [140] achieved a radiochemical conversion (rcc) of 84% within 20 min in DMA at 110 °C under phase-transfer catalysis conditions. The same catalytic system was used by Neumaier, although in that case [^18^F]F^-^ was eluted with Et_4_NHCO_3_ in *^n^*butanol [141]. As demonstrated by the reported examples, the achieved rcc rates were found to be almost quantitative (Table 6, entry 4). The developed reaction conditions were used for the synthesis of a PET probe by means of the radiofluorination of the SnMe_3_ substrate.

**Scheme 34:**
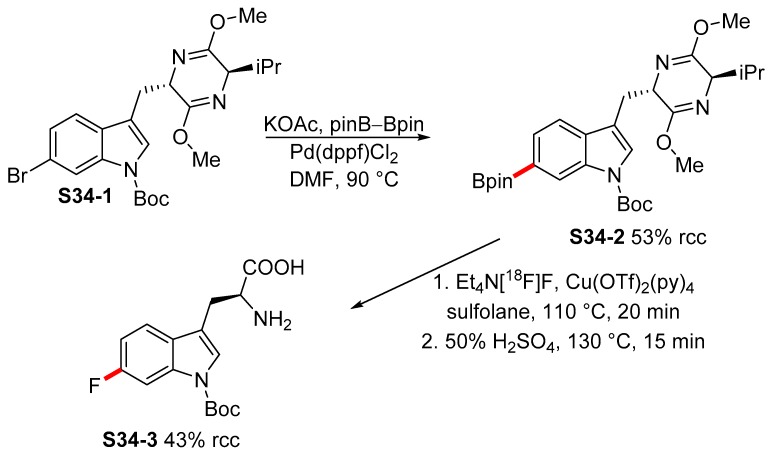
Radiolabeled synthesis of fluorinated L-tryptophan.

Ermert [142] developed a protocol for the copper(II)-catalyzed fluorination of indolylboronic acid pinacol esters by means of Et_4_N[^18^F]F. This protocol was applied for the synthesis of 6-6-[^18^F]fluoro-L-tryptophan (Scheme 34). The starting bromoindole **S34-1** was prepared according to the procedure described in the literature [143]. Then, Miyaura borylation was used for the bromine substitution with the Bpin moiety and the resultant radiolabeled fluorination, along with hydrolysis, afforded the tryptophan derivative **S34-3** in an overall isolated yield of 37% and moderate rcc.

A logical extension of the halogenation reactions of indolylboronic acids can be seen in the case of trifluoromethylation, since a trifluoromethyl group is a prominent structural motif present in biologically active compounds used in the fields of pharmaceuticals and agrochemicals. An early report concerning such a procedure was published by Buchwald [144] (Table 7, entry 1). This copper-mediated oxidative cross-coupling uses TMSCF_3_ (Ruppert’s reagent) as a source of nucleophilic CF_3_ and oxygen as a stoichiometric reoxidant. The isolated yields of trifluoromethylated indoles were 44% and 61%, which demonstrates good agreement with the overall isolated yields. A similar procedure was published by Gooßen (Table 7, entry 2) [145]. This protocol substitutes the inconveniently volatile and moisture-sensitive Ruppert’s reagent for crystalline potassium (trifluoromethyl)trimethoxyborate. Among the other substrates, the isolated yield of 5-(trifluoromethyl)-1-methylindole was only 49%. To overcome the need for stoichiometric amounts of copper, Liu [146] (Table 7, entry 3) suggested the use of (trifluoromethyl)dibenzothiophenium triflate as an electrophilic CF_3_ source. The greatest advantage offered by this method is the tolerance of an unprotected NH group, which meant that an unprotected trifluoromethylated indole was obtained in a 61% yield. Alternatively, Shen [147] exploited the use of Togni’s reagent [148] using a similar method and achieved the highest isolated yield of trifluoromethylated indole of all the discussed procedures (Table 7, entry 4). A slightly different approach to trifluoromethylation was described by Molander et al. (Table 7, entry 5) [149]. They performed the radical trifluoromethylation of organotrifluoroborates by using sodium triflinate (Langlois reagent) as the source of the CF_3_ radicals and five equivalents of *tert*-butyl hydroperoxide (TBHP) in a mixture of solvents. Additionally, the introduction of the trifluoromethoxy group into an indole moiety via the coupling of the corresponding boronic acids was described by Ritter [150]. His two-step, one-pot procedure includes the reaction of indolylboronic acids with NaOH (1 equiv.) and AgPF_6_ (2 equiv.) in methanol, followed by treatment with TAS∙OCF_3_ and the Selectfluor-type reagent (F-TEDA-PF_6_).

**Scheme 35:**
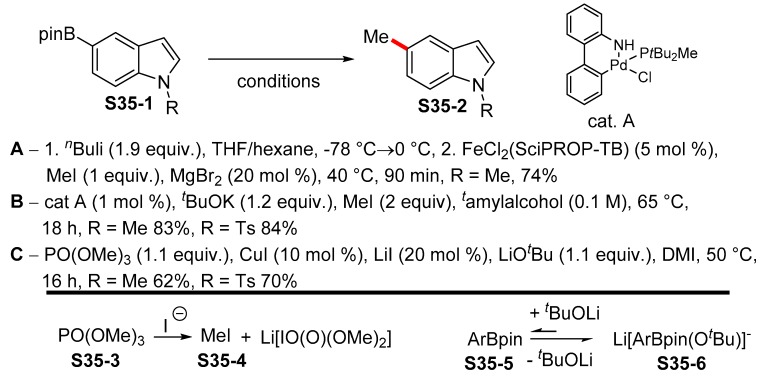
Developed conditions for the methylation of indolylboronic acid pinacol esters.

The methylation of indole boronic esters can be achieved by a simple cross-coupling reaction with methyl iodide when compared to trifluoromethylation. The iron-catalyzed methylation approach described by Nakamura [151] (Scheme 35, condition A) presumably involves a radical mechanism whereby the methylation of the starting compound proceeds in the solvent cage, thereby affording 1,5-dimethylindole in a 74% isolated yield. The presence of a bulky bidentate ligand (SciPROP-TB) is required in this case. More traditional procedures make use of the cross-coupling between indolylboronic acid pinacol esters and methyl iodide catalyzed by palladium catalyst A, as reported by Hartwig et al. (Scheme 35, condition B) [152]. 

The authors provided a simple methylation of indomethacin as an example of the late-stage methylation of a bioactive compound. Alternatively, trimethyl phosphate can be used in combination with iodine salt as a milder source of slow-releasing methyl iodide, as described by Hartwig [153] (Scheme 35, condition C). The isolated yields of methylated indoles were 62–70%. It was proposed that the lithium iodide reacts with the trimethyl phosphate (**S35-3**) to slowly release methyl iodide (**S35-4**). This reaction represents a rate-determining step. Subsequently, the methyl iodide reacts with the arylcopper intermediate so as to give the product of the methylation. Any side reactions are suppressed by the slow release of methyl iodide as well as the acid base reaction between ArBpin **S35-5** and *^t^*BuOLi.

**Scheme 36:**
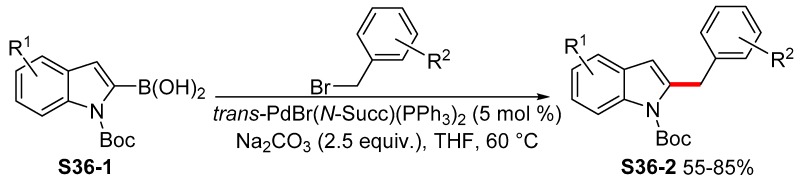
Palladium-catalyzed benzylation of 2-indolylboronic acids **S36-1**.

Gomez [154] focused on the direct benzylation of 2-indolylboronic acids by means of benzyl bromides in the presence of a *trans*-PdBr(N-Succ)(PPh_3_)_2_ catalyst. This catalyst turned out to be the most efficient in terms of suppressing any undesired protodeboronation. The optimized conditions were applied on substituted indoles and benzyl bromides, thereby affording trisubstituted indoles in good isolated yields (Scheme 36). 

A different approach for the synthesis of diarylmethanes was designed by Watson and Garnsey (Table 8, entry 1) [155]. Their methodology started with benzylamines, which were converted into pyridinium salts by refluxing with 2,4,6-triphenylpyrrilium tetrafluoroborate. Then, the benzylation of the indolylboronic acids was achieved by means of an Ni-catalyzed reaction in 1,4-dioxane at 60 °C. The authors hypothesized that the radical mechanism operated along the lines of the developed protocol. This idea was confirmed by the isolation of the benzylated TEMPO radical. The similar nickel-catalyzed benzylation of indolylboronic acid neopentylglycol ester was reported by Tobisu and Chatani (Table 8, entry 2) [156]. The reaction was conducted in toluene at 120 °C in the presence of the NHC ligand. The synthesis of the alkylated indole was performed on a multigram scale in a 90% isolated yield. Analogously to the benzylation protocols, a representative example of the asymmetric allylation of allyl phosphates with boronic acids was described by Hayashi [157]. The allylation of 5-(nepB)-1-methylindole was achieved by means of a CuCl-catalyzed reaction in the presence of sodium methoxide and the *N*-heterocyclic carbene. The formed allylated indole was isolated in an 89% yield and exhibited acceptable enantiomeric access of 88%.

**Scheme 37:**
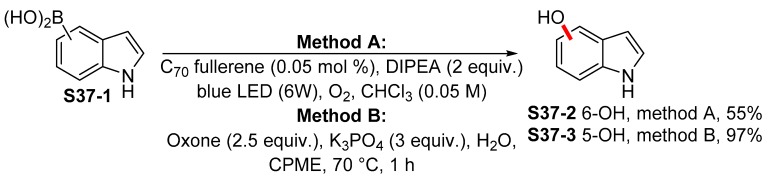
The oxidation of indolylboronic acids as a route to the corresponding phenols.

The hydroxylation of aromatic or heteroaromatic boronic acids represents an alternative reaction pathway for synthesizing the corresponding phenols. In the case of indolylboronic acids or boronates, two protocols have been described in the literature. The first report relies on a C_70_ fullerene-catalyzed reaction under photocatalytic conditions (Scheme 37, method A) [158]. However, the isolated yield of 6-hydroxyindoles **S37-2** is significantly lower when compared to the tested aromatic boronic acids. A quantitative yield of 5-hydroxyindole was achieved by means of oxone oxidation under phase-transfer conditions (Scheme 37, method B) [159]. In addition to the quantitative yield of **S37-3**, the described protocol allows for the chemoselective oxidation of boronic acids over pinacol boronates. The chemoselectivity is achieved via the faster trihydroxyboronate transfer from the organic layer to the water layer.

In an effort to expand the diversity-oriented synthesis of substituted indoles, two different carbonylation procedures have been reported (Table 9, entries 1 and 2). In the first report, Skrydstrup [160] made use of a traditional palladium catalyst with a Xantphos ligand and the carbonylation of boronic acids with CO and *N*,*N*-diethyl-α-bromo-α,α-difluoroacetamide. It was determined that the reaction is suitable for the carbonylation of unprotected indoles, thereby affording the product in a good isolated yield. 

The reaction was also extended to include α-bromo-α,α-difluoroacetates. The presence of two equivalents of 1,4-dinitrobenzene suppressed the reaction, while the two equivalents of TEMPO resulted in the isolation of the corresponding adducts. These experiments indicated a SET mechanism for the carbonylation. Different from Skrydstrup’s protocol, Szostak [161] applied an alternative method for the carbonylation of boronic acids. His protocol is based on the cross-coupling reaction of the activated C−N amide bond. Thus, the cyclic amide **T9-3** was found to be the best substrate, affording various ketones along with the indole in good isolated yields (Table 9, entry 2). The methodology of the CO carbonylation of indolylboronic acid pinacol boronic esters was extended to cover methoxycarbonylation (Table 9, entry 3) [162]. Weinreb amides are useful synthons for Weinreb ketone synthesis [163]. Therefore, it is unsurprising that the simple and high-yielding synthesis of Weinreb amides has been pursued (Table 9, entry 4) [164]. Different boronic acids and organotrifluoroborates were mixed with *N*-methoxy-*N*-methylcarbamoyl chloride in the presence of PdCl_2_(PPh_3_)_2_ and potassium phosphate in ethanol, thereby affording, for instance, 1*H* protected and unprotected indole-based amides in good isolated yields.

Among the other aromatic and heteroaromatic boronates, 1-methyl-5-nepBindole smoothly reacted with arylcarbamate in the rhodium-catalyzed reaction so as to yield 5-substituted indole in a 93% isolated yield (Table 10, entry 1). Interestingly, the cross-coupling reaction was achieved by means of a catalytic amount of the Rh catalyst [165]. The Liebeskind−Srogl cross-coupling reaction was used for the synthesis of substituted BODIPY involving Biellmann BODIPY (Table 10, entry 2) [166]. The reaction was catalyzed by Pd_2_(dba)_3_ in the presence of a catalytic amount of trifurylphosphine and a stoichiometric amount of CuTC, thereby leading to variously substituted BODIPYs. The reaction can also be performed under microwave heating, and the fluorescent efficiency was tested on prepared substances. 

**Scheme 38:**
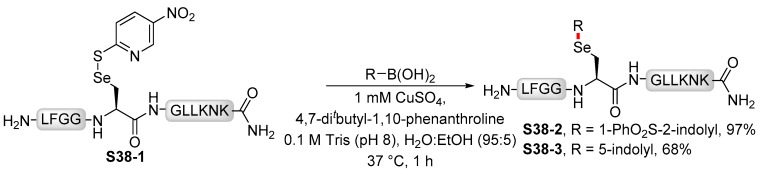
An umpolung approach for the chemoselective arylation of unprotected peptides.

A similar type of cross-coupling reaction was described by Pentelute and Buchwald (Scheme 38) [167]. In their report, they applied an umpolung strategy for the bioconjugation of the selenocysteine in unprotected peptides. The reaction was conducted by mixing boronic acids in the presence of copper (II) sulfate and the phenanthroline ligand. The incorporation of diverse indolylboronic acids **S38-2** and **S38-3** was achieved in good yields.

**Scheme 39:**
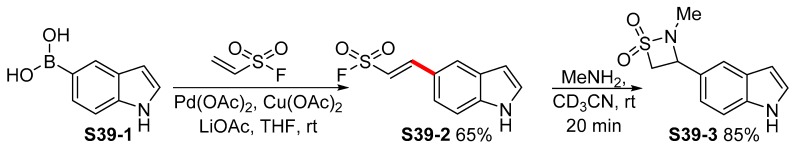
Oxidative boron Heck coupling of indolylboronic acids for the synthesis of sulfonylfluoride and β-sultam.

Arvidsson et al. [168]. developed a general methodology for the synthesis of substituted β-aryl ethenesulfonyl fluorides by means of oxidative Heck coupling. The optimized ligandless reaction conditions involved copper(II) acetate as the oxidant and THF as the solvent. The reaction was applied to the synthesis of 5-substituted indole and the following reaction with methylamine resulted in β-sultam **S39-3** in an 85% isolated yield (Scheme 39).

**Scheme 40:**
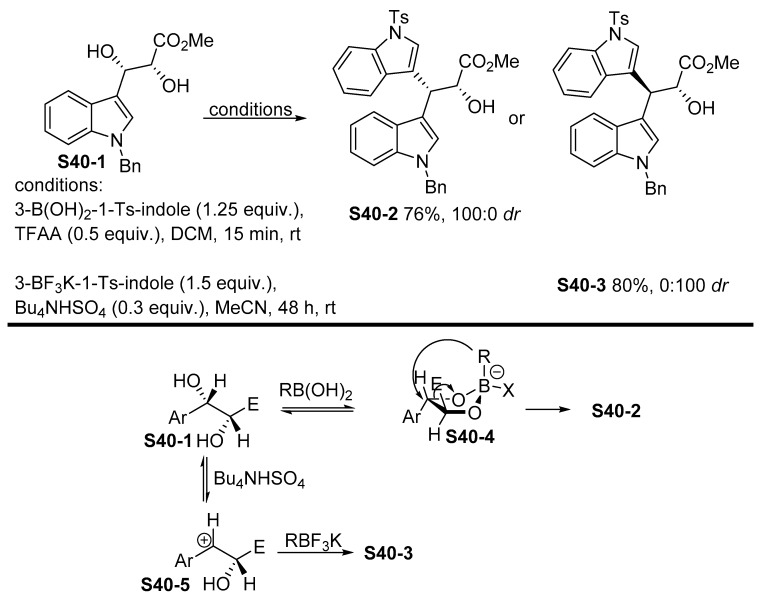
A transition-metal-free diastereoselective reaction between vicinal diols and boronic acid derivatives.

The conditions necessary for a transition-metal-free reaction between electron-rich aryl/hetarylboronic acids or trifluoroborates and unprotected vicinal diols have also been published (Scheme 40) [169]. Two different reaction conditions were developed in order to gain opposite stereochemical outcomes. Trifluoroacetic anhydride preferentially formed the substituted indole **S40-2** in a 76% isolated yield as a single diastereomer upon reaction with boronic acid. 

Alternatively, Bu_4_NHSO_4_ along with organotrifluoroborate gave the pure diastereomer **S40-3** in an 80% yield. Based on the experimental evidence, it is expected that the reaction proceeds according to two different types of alcohol **S40-1** activations. In the case of the boronic acids, the formation of a cyclic ester is preferred, leading to the product **S40-4**, which in turn forms the product **S40-2**. Meanwhile, Bu_4_NHSO_4_ facilitates the formation of carbocation **S40-5**, resulting in the formation of the opposite diastereomer.

**Scheme 41:**
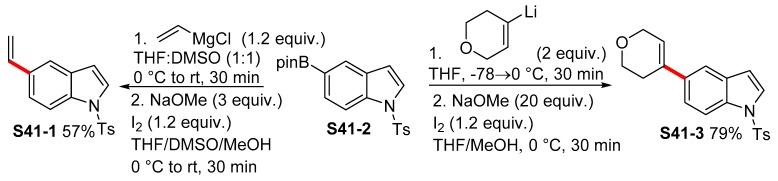
The Zweifel coupling of 5-indolylboronic pinacol boronic acid esters.

A simple approach to the synthesis of substituted indoles was developed by Aggarwal (Scheme 41) [170]. The protocol starts with easily available pinacol boronic esters, which are combined with vinylmagnesium chlorides or vinyllithiums. The formed ate complexes are transformed into the final product via the use of iodine and sodium methoxide. The synthesis of the substituted indoles **S41-1** and **S41-3** in acceptable yields is achieved.

**Scheme 42:**
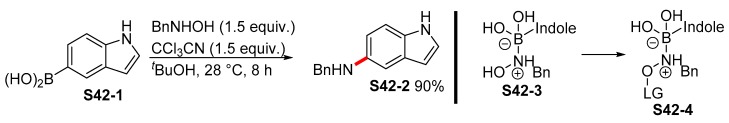
Transition-metal-free amination of indolylboronic acids.

In 2018, Niu et al. [171] developed a simple protocol for the transition-metal-free amination of boronic acids. The authors proposed that the initially formed ate complex **S42-3** requires hydroxyl group activation so as to accomplish the amination (Scheme 42). Indeed, the activation was achieved with trichloroacetonitrile in *^t^*butanol. Thus, the synthesis of 5-(*N*-benzylamino)-1*H*-indole was completed in a 90% isolated yield. Aside from the other aromatic and heteroaromatic substrates, the same methodology was used for the amination of a tripeptide, a steroidal moiety and a protected carbohydrate showing excellent functional group tolerance. In addition to the aforementioned transition-metal-free amination of boronic acids, the copper(II)-acetate-catalyzed oxidative coupling of 1H-pyrazolo[3–*b*]pyridine-amine and 2-aminobenzimidazoles has been described in the literature [172]. 

### 3.2. Cyclization Reactions of Indolylboronic Acid Derivatives

**Scheme 43:**
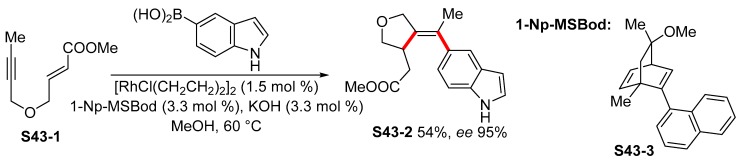
The formation of 3,4-disubstitutedtetrahydrofurans via the rhodium-catalyzed cyclization of the ether **S43-1**.

Several cyclizations of diverse substrates have been developed covering both indolylboronic acids and transition-metal catalysis. In 2019, the Darses research group developed a methodology for the asymmetric cyclization of (allyl)propargylether catalyzed by a rhodium catalyst (Scheme 43) [173]. The reaction is performed in methanol at 60 °C, and the reaction outcome depends on the structure of the ligand. When conducted in the presence of 1-Np-MSBod (**S43-3**), the exclusive formation of 3,4-disubstitutedtetrahydrofurans was observed in 47−60% isolated yields. The indolyl derivative **S43-2** was isolated in a 54% yield and 95% *ee*. The use of the similar bicyclo[2.2.2]octa-2,5-diene ligand led to tetrahydrobenzo[*d*]ixepines in a similar yield and *ee*. 

**Scheme 44:**
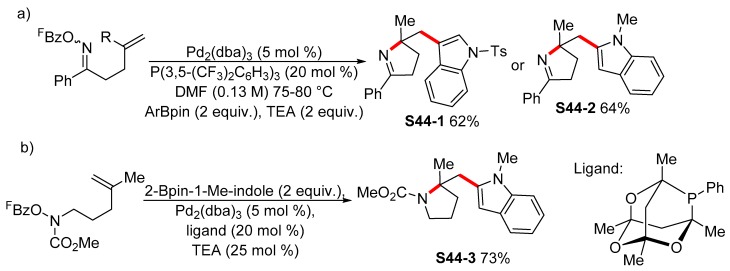
Palladium-catalyzed cyclization via O−N bond activation.

As demonstrated by Bower, palladium-catalyzed cyclizations of oxime esters or *N*-(pentafluorobenzoyloxy)carbamates have been successfully used to synthesize trisubstituted 1-pyrrolines [174] (Scheme 44a) and pyrrolidine [175] (Scheme 44b). Both strategies take advantage of the *N*-pentafluorobenzoyloxy group, which activates the N−O bond for the oxidative addition of palladium, although it requires two different ligands. The catalyzed alkene 1,2-carboamination reaction requires the tris[3,5-bis(trifluoromethyl)phenyl]phosphine ligand (Scheme 44a), while the aza-Heck cyclization relies on an adamantyl-type ligand (Scheme 44b) to give good isolated yields of the products **S44-1−S44-3**.

**Scheme 45:**
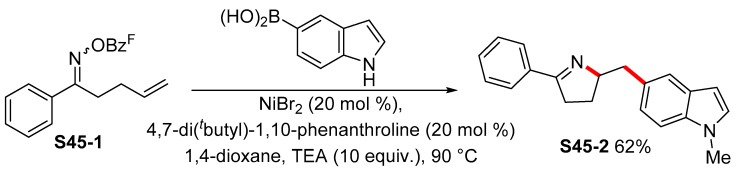
Nickel-catalyzed cyclization of the activated oximes **S45-1** in the presence of indolylboronic acid.

Analogously to Bower’s work, Selander et al. developed the 1,2-aminoarylation of oxime ester-tethered alkenes catalyzed by a nickel catalyst along with the phenanthroline ligand, thereby affording 2,5-disubstituted-1-pyrrolines (Scheme 45) [176]. The cyclization reaction showed a good substrate scope with isolated yields ranging from 38−82%. The indolylboronic acid gave the product **S45-2** in a 62% isolated yield. The authors proposed the cleavage of the N−O bond by means of SET, with the subsequent cyclization proceeding via a radical addition to the double bond. This assumption was confirmed by trapping the radical intermediate with TEMPO. The developed conditions were applied to the synthesis of ABI-274, a potent tubulin inhibitor effective in relation to multidrug-resistant cancer cells.

**Scheme 46:**
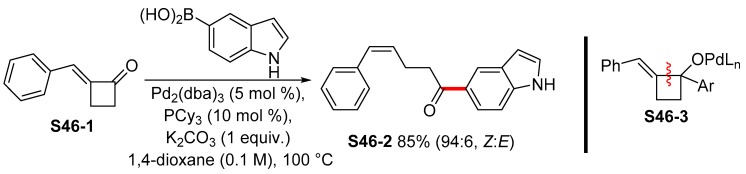
Palladium-catalyzed ring expansion of cyclobutanone en route to Z-disubstituted alkenes.

There exists an inverse approach to substituted indoles based on a cyclobutanone ring expansion (Scheme 46) [177]. In this case, the substituted cyclobutanone **S46-1** was treated with 5-indolylboronic acid in the presence of a palladium precatalyst and the tricyclohexylphosphine ligand. The corresponding 5-substituted indole was formed in a high isolated yield and an excellent *Z*:*E* ratio. The methodology also tolerates other substituted phenyl, styrenyl, and heteroarylboronic acids. Based on the deuterium experiments, the formation of the palladium complex **S46-3** by means of 1,2-addition is expected as a key reaction intermediate. The subsequent β-carbon elimination via C_sp3_−C_sp2_ bond cleavage and protolysis is responsible for the formation of the final product. 

### 3.3. Multicomponent Reactions of Indolylboronic Acid Derivatives

**Scheme 47:**
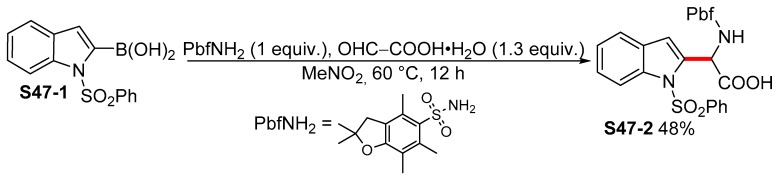
Three-component reaction for the preparation of α-substituted glycines.

Multicomponent reactions can significantly facilitate the preparation of complex structures of organic molecules due to lowering the required number of reaction steps. The use of boronic acids is widespread in such reactions, as the creation of the quaternary borate salt known as the “ate” complex enables the transfer of the boron substituent onto, for example, an imine – as is the case in the Petasis-Borono-Mannich multicomponent reaction. The classical metal-free Petasis reaction is the topic of the work published by Manolikakes [178] (Scheme 47). He used sulfonamides as the amine component in a three-component reaction with a glyoxylic and a boronic acid. This way, the medicinally important sulfonamide group was introduced into various α-substituted glycines, although the yield of the indole-based substrate **S47-2** was only 48%. The sulfonamide group in the prepared compounds could serve as a protecting group and a possible site of alteration for modulating the biological properties and increasing the metabolic stability of the compound.

**Scheme 48:**
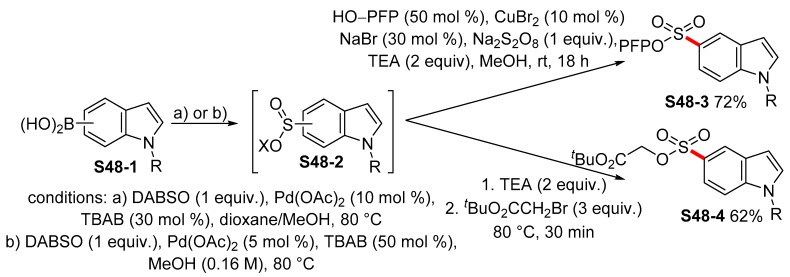
The synthesis of sulfones and sulfonate esters by means of DABSO.

A more general methodology for the synthesis of sulfones, sulfonamides [179], and sulfonate esters [180] was developed by Willis et al. (Scheme 48). In their reports, the synthesis of the target compounds is realized via the conversion of aryl/hetarylboronic acid, for example, indolylboronic acid **S48-1**, into the sulfonates **S48-2**. The reaction is carried out in the presence of the sulfur dioxide surrogate 1,4-diazabicyclo[2.2.2]octane bis(sulfur dioxide) (DABSO) and a Pd-catalyst. The direct conversion of the sulfinate **S48-2** into the sulfones **S48-4** is performed under transition-metal-free conditions by means of *tert*-butyl α-bromoacetate, while the conversion into the sulfonate esters **S48-3** is mediated by copper(II) bromide. A similar direct copper-catalyzed sulfonamides synthesis was reported by the same authors [181]. Primary amines and sterically demanding secondary amines are not tolerated in this reaction. The authors also incorporated several pharmaceutically important amines into the structures of the indole-5-sulfonamides so as to display the potential value of this method in relation to drug design and discovery.

**Scheme 49:**
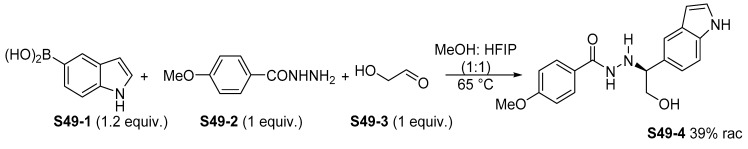
The Petasis reaction of indolylboronic acids.

In 2012, Nielsen [182] described a three-component coupling reaction involving α-hydroxy aldehydes and boronic acids (Scheme 49) in a mixture of solvents, namely MeOH:hexafluoroisopropanol (HFIP). The products formed in the Petasis reaction varied from 26–95% in terms of the yield. The hydrazido alcohols **S49-4** obtained using this protocol were subjected to a cyclization reaction in relation to the corresponding oxadiazolones and oxazolidinones, that is the target compounds of the work due to their possible antibacterial activity. Later on, the same authors [183] reported a four-component Petasis-type reaction that was used for the synthesis of dioxadiazaborocines. 

**Scheme 50:**
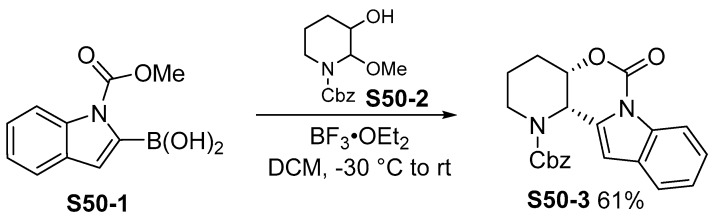
A Petasis-type reaction for the preparation of *cis*-2-aryl-3-hydroxypiperidines **S50-3**.

A Petasis-type reaction was utilized by Onomura [184] when designing a versatile procedure for the diasterocontrolled synthesis of *cis*-2,3-disubstitutedpiperidine **S50-3** (Scheme 50). The Lewis-acid-catalyzed reaction of indolyl boronic acid **S50-1** with cyclic *N*,*O*-acetal **S50-2** provided the cyclic carbamate **S50-3**.

**Scheme 51:**
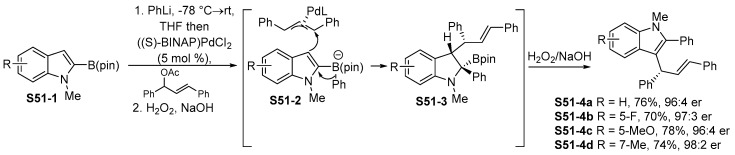
The procedure for the preparation of C2- and C3-substituted indoles.

In 2017, Ready [185] reported on the three-component palladium-catalyzed coupling of indoles with boronic esters and allylic acetates. The multicomponent reaction generates ate complexes **S51-2** via the reaction of indolylboronic acid pinacol esters **S51-1** (Scheme 51). Then, the formed ate complexes **S51-2** are rearranged into 2,3-dihydroindoles **S51-3**. Overall, the isolated yields of the disubstituted indoles **S51-4a−S51-4d** are high, thereby demonstrating high diastereomeric and enantiomeric ratios. 

**Figure 2:**
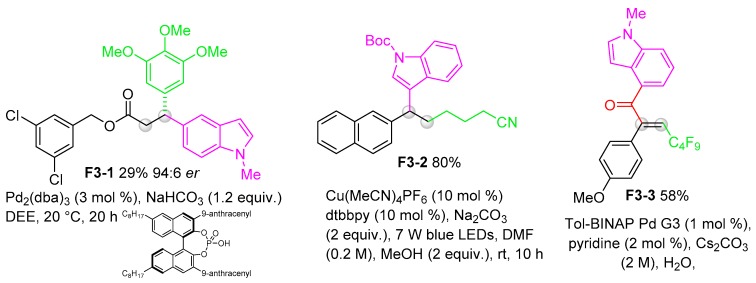
The structures of substances prepared by means of the multicomponent reactions of indolylboronic acids.

The other multicomponent reactions include indolylboronic acids are summarized in Figure 2. The palladium-catalyzed multicomponent reaction [186] of indole boronic acids includes the 1,1-diarylation of terminal alkenes by means of boronic acids and aryldiazonium salts. This method is highly enantioselective due to the utilization of a chiral anion phase-transfer catalyst, and the indole-based product **F3-1** was obtained in a 29% isolated yield and an excellent enantiomeric ratio. 

Another possibility with regard to 1,2-diarylation, as published by Xiao [187], employs nitrogen-centered radicals created from cyclobutanone oxime derivatives. The reaction is carried out in the presence of a copper(I) catalyst and with 4,4′-di-*tert*-butyl-2,2′-dipyridyl (dtbbpy) serving as the ligand. This multicomponent reaction is able to accommodate the 3-indolyl moiety, affording the product **F3-2** in an excellent isolated yield. A related procedure concerning the 1,2-addition of boronic esters and aryl triflate to vinyl magnesium chloride was published by Morken [188]. The stereoselective preparation of trisubstituted alkenes with perfluoroalkyl chains was also described [189]. This four-component protocol makes use of carbon monoxide, terminal alkynes, perfluoralkyliodide, and arylboronic acids. A trisubstituted alkene **F3-3** was prepared in a moderate yield, while the overall isolated yields ranged from 43−90%. The double-bond configuration was determined by the through space ^13^C−^19^F coupling.

### 3.4. Addition of Indolylboronic Acids or Boronates to Multiple Bonds

The 1,4-addition of aryl- or hetarylboronic acids can be catalyzed by transition metals. Both published procedures (Table 11, entries 1 and 2) are catalyzed by a rhodium catalyst under identical reaction conditions. 

Viaud-Massuard [190] developed the asymmetric addition on 7-azaindoles as an example of an exocyclic benzylidene lactamyl Michael acceptor. The reaction was performed in the presence of the (*R*,*R*)-Phbod ligand, which ensured the 94% ee of the indole-based product (Table 11, entry 1). A ligandless condition was described by Howell et al. (Table 11, entry 2) [191]. They studied the Michael addition of boronic acids onto α-methylene-β-lactones under similar reaction conditions [190]. Generally speaking, the reaction provided a mixture of diastereomers, including β-propiolactone substituted by a 1-methyl-5-indolyl substituent. The synthesis of nocardiolactone was also described in this paper. The palladium-catalyzed γ-selective hydroarylation of a β,γ-unsaturated secondary amide was reported by Engle (Table 11, entry 3) [192]. In his report, palladium acetate was used to catalyze a regioselective hydroarylation with arylboronic acids, although the substrate scope was limited to amides bearing the *N*-8-aminoquinoline (AQ) substituent. Based on mechanistic studies, it was proposed that palladium coordinates to the AQ moiety, and further, that transmetalation represents the rate-controlling step. In a different paper, Scarso et al. studied the course of the conjugated addition of boronic acids to vinylidenebisphosphonate (VBP) [193]. This reaction represents a valuable contribution to the synthesis of *gem*-bisphosphonates. The reaction takes advantage of a simple copper(II) catalyst and tolerates diverse boronic acids, including 4-indolylboronic acids, which afforded the expected product in an 83% isolated yield (Table 11, entry 4). The same catalytic system is able to facilitate the Friedel–Crafts reaction between VBP and indoles, thereby expanding the scope for the preparation of *gem*-bisphosphonates. The regio- and stereoselective hydroarylation of propargylic carbamates by means of aryl- heteroarylboronic acids catalyzed by a Ni(cod)_2_ catalyst was reported by Jarvo (Table 11, entry 5) [194]. The rac-2-(di-*tert*-butylphosphino)-1,1′-binaphthyl (TrixiePhos) was determined to be the ligand of choice in terms of maintaining the quantitative conversion of the starting alkynes, along with excellent regio- and stereoselectivity. The isolated yields of the trisubstituted alkenes were good, which is illustrated by the provided example (Table 11, entry 5). The methodology was later extended to include the formal synthesis of tamoxifen.

**Scheme 52:**
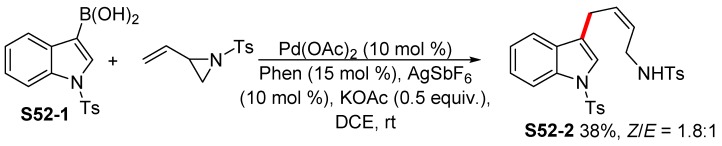
Palladium-catalyzed hydroarylation of vinylaziridines.

Recently, Hyland described a different kind of (*Z*)-selective palladium-catalyzed addition of aryl boronic acids to vinylaziridines (Scheme 52) [195]. This reaction makes use of palladium acetate as a catalyst in combination with the phenanthroline ligand and AgSbF_6_. Although the reaction is said to be (*Z*)-selective, however the reported *Z*:*E* ratio varies between 1.6:1 and 4.5:1. This reactivity is demonstrated by the reaction of indolylboronic acid **S52-1**, which gave a 38% yield of the substituted indole **S52-2** as a mixture of *Z*:*E* isomers in a ratio of 1.8:1.

**Scheme 53:**
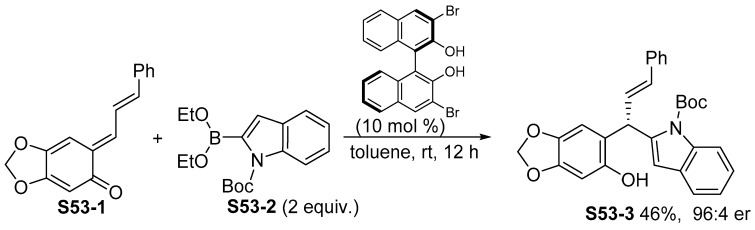
Enantioselective addition of indolylboronic acid diethyl esters to *o*-quinone methide catalyzed by chiral biphenol.

Organocatalytic additions are considered to be more environmentally acceptable than transition-metal-catalyzed reactions. However, practical examples concerning a series of indolylboronic acids can only be found in three reports. In 2012, Schaus described the enantioselective addition of diethyl boronates to *o*-quinone methides **S53-1** (Scheme 53) [196]. The reaction is catalyzed by 10 mol % of the BINOL ligand in toluene at room temperature. Aromatic and heteroaromatic boronates provide a high yield (46−85%) with regard to aromatic phenols, in addition to an excellent enantiomeric ratio of ≥90:10. In the case of indolylboronate **S53-2**, the isolated yield of the product **S53-3** was only 46% and the enantiomeric ratio was 96:4. Moreover, the published protocol was applied to the short synthesis of (*S*)-4-methoxydalbergione.

**Scheme 54:**
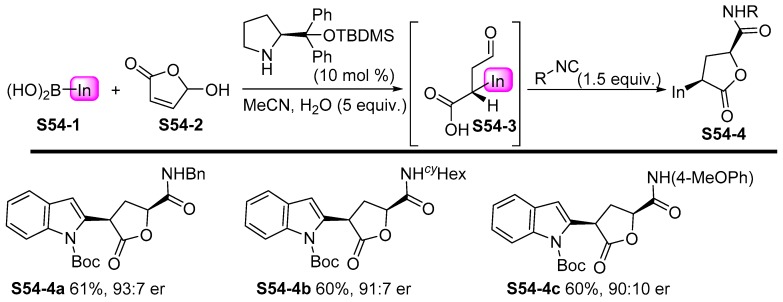
Organocatalytic addition of indolylboronic acids and the intramolecular Passerini reaction for the synthesis of cyclic lactones.

A combination of the organocatalytic Michael addition of boronic acids and the diastereoselective intramolecular Passerini reaction was used for the synthesis of chiral γ-lactones **S54-4** (Scheme 54) [197,198]. This one-pot sequential synthesis starts with the reaction between the Boc-protected 2-indolylboronic acids **S54-1** and 5-hydroxyfuran-2(5*H*)-one (**S54-2**) in the presence of a diphenylprolinol catalyst. The organocatalyst activates the starting 5-hydroxyfuran-2(5H)-one, while the subsequent addition of indolylboronic acid **S54-1** provides the acyclic intermediate **S54-3**, which undergoes a diastereoselective Passerini reaction with isonitriles. A series of chiral lactones **S54-4a**−**S54-4c** substituted with the indole moiety were prepared using this methodology.

In addition to the above-mentioned additions of diverse boronic acids to activated and non-activated double bonds, more traditional 1,2-addition reactions with C-heteroatom double bonds have been described. Thus, the organocatalytic formylation of boronic acids with glyoxylic acid [199], the palladium-imidazolinium carbene-catalyzed arylation of aldehydes with boronic acids [200], the rhodium-catalyzed addition of boronic acids to 2,2-disubstituted malononitriles [201,202], the rhodium-catalyzed addition of boronic acids to α-ketoesters [203], and the addition of boronic acids to nitrosoarenes [204] are all valuable examples. 

## 4. Conclusions

The aforementioned examples of transition-metal-catalyzed cross-coupling reactions, multicomponent reactions, addition reactions, and transition-metal-free alternatives, document tremendous progress in relation to the synthetic applications of indolylboronic acids and aryl boronic acid in general. This progress can be illustrated by over 4000 patent applications describing chemical transformations of indolylboronic acid derivatives. Significant progress has been made in the area of indolylboronic acids synthesis via transition-metal-catalyzed C−H borylations, Miyaura borylation, and *ortho*-substituted aniline cyclizations. Also transition-metal-catalyzed and transition-metal-free reactions of indolylboronic acid derivatives were intensively studied in the past. It can be expected, that more publications concerning a cooperative catalysis of indolylboronic acids will emerge soon as examples from new area of transition-metal-catalyzed reactions. Moreover, increased attention should focus on multicomponent reactions of indolylboronic acids as a simple and efficient way to complex indole-based molecules. Thus, the discovery of novel transformations and reactions on the part of indole boronates or boronic acids will undoubtedly widen the pool of modified indoles, which may find interesting practical applications in the near future.

## Figures and Tables

**Table 1:** The preparation of indolylboronates by means of Miyaura borylation.

	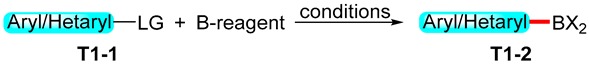			
**Entry**	**LG**	**B-reagent**	**Conditions**	**T1-2**
1	I	pinB-Bdan (2 equiv.)	Pd_2_(dba)_3_ (5 mol %), Cs_2_CO_3_, MeOH, reflux	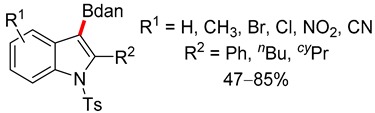
2	Cl	H_2_B-N*^i^*Pr_2_	Pd(OAc)_2_ (2 mol %), XPhos (6 mol %), KI (2 mol %), TEA, EtOAc, 50 °C, 16 h	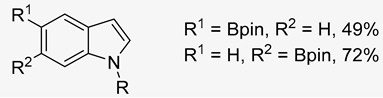
3	Br	H-Bpin (2.5 equiv.)	CuI (10 mol %), Fe(acac)_3_ (10 mol %), TMEDA (1.5 equiv.), NaH (1.5 equiv.), -10 °C, 18 h	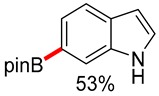
4	I, Br	pinB-Bpin	[Cu(DPEphos)(DMEGqu)]PF_6_ (2.5 mol %) DIPEA (1.2 equiv.), pyridine (20 mol %), MeCN/H_2_O, 12 h blue LED	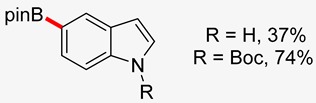
5	Br	pinB-Bpin (1.3 equiv.)	Co(IMes)_2_Cl_2_ (2.5 mol %), KOMe (1.3 equiv.), MTBE, 50 °C, 8 h	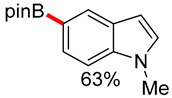
6	F	H-Bpin (2 equiv.)	CoCl_2_ (10 mol %), L1 (15 mol %), Mg (1.5 equiv.), THF, 60 °C, 16 h	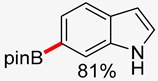
7	F	nepB-Bnep (3 equiv.)	Ni(cod)_2_ (5 mol %), PCy_3_ (20 mol %), NaOPh (3 equiv.), THF, 110 °C	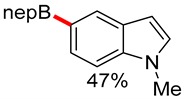
8	CN	nepB-Bnep (2 equiv.)	[RhCl(cod)]_2_ (5 mol %), Xantphos (20 mol %), DABCO (1.0 equiv.), toluene, 100 °C, 15 h	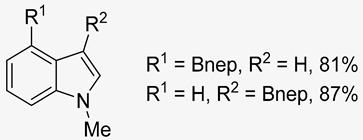
9	Cl	pinB-Bpin	hν, TMEDA, TBAF, MeCN/H_2_O, acetone	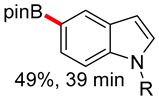
10	Br	PhMe_2_Si-Bpin (1.5 equiv.)	KOMe (1.2 equiv.), DME, 30 °C, 1 h	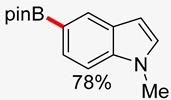

**Table 2:** Decarboxylative cross-coupling for the synthesis of indolylboronic acids.

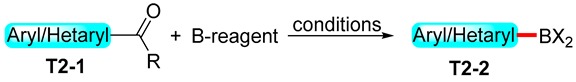			
**Entry**	**R**	**Conditions**	**T2-2**
1	OH	pinB−Bpin (1.5 equiv.), Pd(OAc)_2_, dppb (10 mol %), TEA, piv_2_O, dioxane 160 °C, 15 h	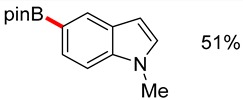
2	SEt	pinB−Bpin (2 equiv.), [Rh(OH)(cod)]_2_ (5 mol %), P(*^n^*Bu)_3_ (50 mol %), KOAc (20 mol %), CPME, 80 °C, 24 h	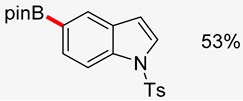
3	ONPhth	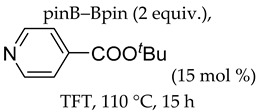	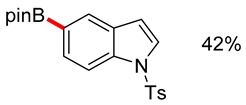
4	ONPhth	pinB−Bpin (3.5 equiv.), pyridine (1.5 equiv.) Cs_2_CO_3_ (0.5 equiv.), EtOAc, 35 °C, 400 nm, 24 h	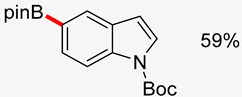

**Table 3:** The synthesis of indolylboronic acids or esters via the cyclization of substituted anilines.

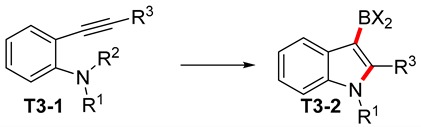			
**Entry**	**R^1^, R^2^**	**Conditions**	**T3-2**
1	H, Ts	BCl_3_ (2 equiv.), DCM rt, then pinacol, TEA, rt 1 h	36−83%, R^3^ = Ph, 4-MePh, 4-MeOPh, 3-thienyl, 2-naphthyl, etc.
2	R^1^ = cat, dan R^2^ = Me or Bn	B(C_6_F_5_)_3_ (5 mol %)	42−60%, R^3^ = Ph, 4-BrPh, 4-*^t^*BuPh, *^n^*Bu, ferrocenyl
3	R^1^ = cat R^2^ = Mbs, Ts	IPrAuTFA (5 mol %), NaTFA (20 mol %), toluene, 80 °C, 20 h then pinacol (3 equiv.), THF, rt, 1 h	55−80%, R^3^ = Ph, 2-thienyl, *^n^*Bu, *^cy^*Pr, 2-BrPh, TBDPSOCH_2_, etc
4	R^1^ = Ts, Ms R^2^ = H	pinB-Bpin (2 equiv.), Pd_2_dba_3_ (5 mol %), AsPh_3_ (10 mol %), Cs_2_CO_3_, DMA, 60 °C, 30 min.	51−79%, R^3^ = Ph, Bu, BnOCH_2_, etc
5	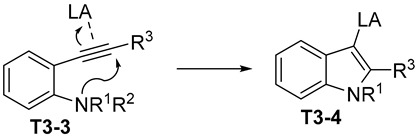		

**Table 4:** Lewis-acid-catalyzed borylation of indoles and electron-rich arenes.

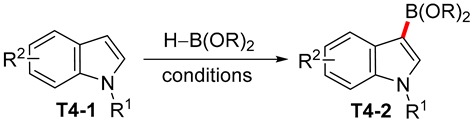		
**Entry**	**Conditions**	**T4-2**
1	H-Bcat (0.55 equiv.) B(C_6_F_5_)_3_ (5 mol %), C_6_D_6_, rt, 2 h	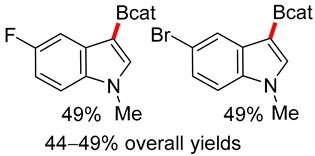
2	H-Bcat (4 equiv.) B(C_6_F_5_)_3_ (10 mol %), toluene, 120 °C, 72 h 2. pinacol (3 equiv.), TEA, rt, 1 h	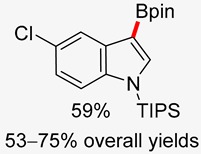
3	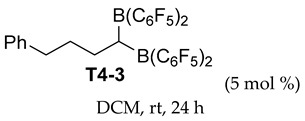	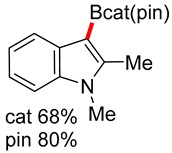

**Table 5:** Simple transition-metal-catalyzed modifications of indolylboronic acids.

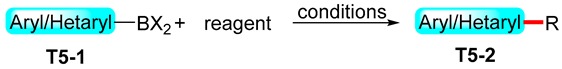			
**Entry**	**T5-1**	**Conditions**	**R, yield (%)**
1	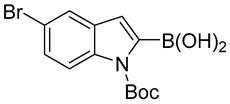	Ph_3_PAuNTf_2_ (5 mol %), dimethyl carbonate, μW, H_2_O (10 equiv.), 90 °C, 1 h	−H, 73
2	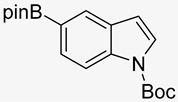	NaN_3_ (1.5 equiv.), Cu(OAc)_2_ (0.1 equiv.), MeOH (0.2 M), 55 °C, 24 h	−N_3_, 51
3	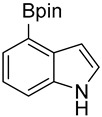	[^11^C]NH_4_CN/NH_3_, PdCl_2_(PPh_3_)_2_, K_2_CO_3_, DMF, 100 °C, 5 min	−^11^CN, 90 ± 15 (rcy)

**Table 6:** The halogenation of indolylboronic acids.

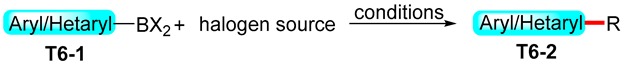			
**Entry**	**T6-1**	**Halogen source, conditions**	**T6-2**
1	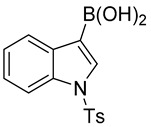	I_2_ (1 equiv.), KF (3.3 equiv.) 1,4-dioxane (0.1 M), 80 °C, 1 h	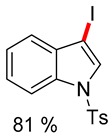
2	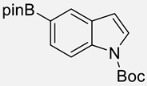	(*^t^*BuCN)_2_CuOTf (2 equiv.), [Me_3_py]PF_6_ (3 equiv.), AgF (2 equiv.), THF, 50 °C, 18 h	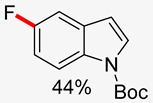
3	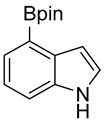	[^18^F]F^-^, trifluoromethanesulfonate, Cu(OTf)_2_Py_4_, DMA, air, 110 °C, 20 min	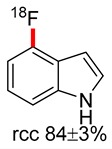
4	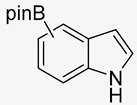	(i) elution of [^18^F]F^-^ with Et_4_NHCO_3_ in *^n^*BuOH (ii) [Cu(OTf)_2_(py)_4_], DMA, 110 °C, 20 min, air	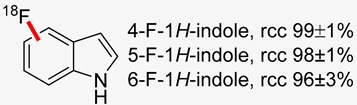

**Table 7:** Examples of indole trifluoromethylation.

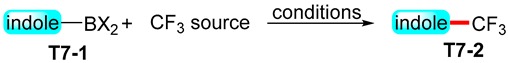			
**Entry**	**BX_2_**	**[CF_3_], conditions**	**T7-2**
1	B(OH)_2_	TMSCF_3_ (2 equiv.), Cu(OAc)_2_ (1 equiv.), 1,10-phenanthroline (1.1 equiv.), CsF (2 equiv.), O_2_, *^i^*PrCN, DCE, 4 Å MS, rt	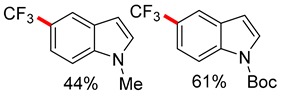
2	Bpin	K[CF_3_B(OMe)_3_] (2 equiv.), Cu(OAc)_2_ (1 equiv.), O_2_,DMSO, 60 °C	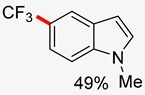
3	B(OH)_2_	(trifluoromethyl)dibenzothiophenium triflate, CuOAc (20 mol %), 2,4,6-trimethylpyridine (2 equiv.), DMAC, 0 °C	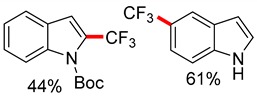
4	B(OH)_2_	Togni’s reagent, CuI (2 mol %), phenanthroline, K_2_CO_3_ (2 equiv.), 35 °C, DME	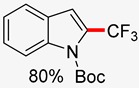
5	BF_3_K	NaSO_2_CF_3_ (3 equiv.), TBHP (5 equiv.), CuCl (1 equiv.), DCM/MeOH/H_2_O, rt	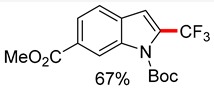

**Table 8:** The benzylation of indolylboronic acids.

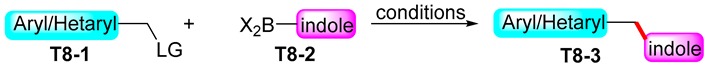				
**Entry**	**LG**	**BX_2_**	**Conditions**	**T8-3**
1	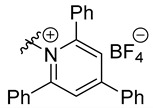	B(OH)_2_	PhenNi(OAc)_2_∙xH_2_O (5 mol %), K_3_PO_4_ (3.4 equiv.), EtOH (5 equiv), dioxane (0.5 M), 60 °C, 5 h	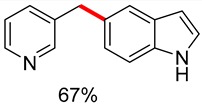
2	OMe	Bnep	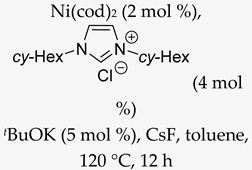	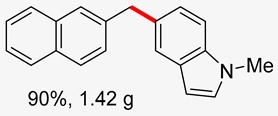

**Table 9:** Carbonylation of indolylboronic acids.

	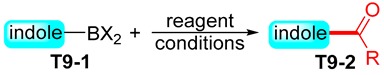			
**Entry**	**BX_2_**	**Reagent**	**Conditions**	**T9-2**
1	Bpin	BrF_2_CCONEt_2_ (2 equiv.), CO	Pd(PPh)_4_ (5 mol %), Xantphos (10 mol %), K_2_CO_3_ (4 equiv.), toluene/H_2_O (9:1), 80 °C	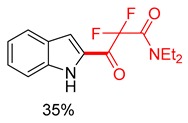
2	B(OH)_2_	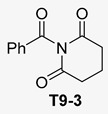	Pd(OAc)_2_ (3 mol %), PCy_3_HBF_4_ (12 mol %), K_2_CO_3_, H_3_BO_3_, THF, 65 °C, 15 h	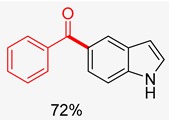
3	Bpin	CO (1 atm)	Pd(OAc)_2_/2PPh_3_ (5 mol%), pbq (1 equiv.), MeOH, rt, 18 h	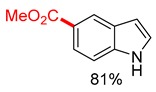
4	B(OH)_2_	ClCO−NMe(OMe)	PdCl_2_(PPh_3_)_2_, K_3_PO_4_∙H_2_O, EtOH, 65 °C	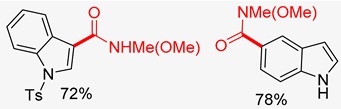

**Table 10:** Palladium- and rhodium-catalyzed arylation of indolylboronic acids.

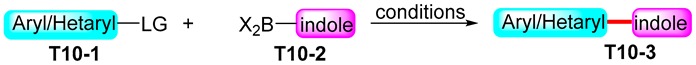				
**Entry**	**LG**	**BX_2_**	**Conditions**	**T10-3**
	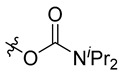	Bnep	[RhCl(cod)] (5 mol %), I(2Ad)∙HCl (20 mol %), NaOEt (2 equiv.), toluene, 130 °C, 20 h	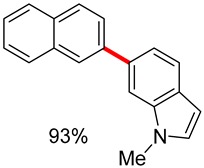
	-SMe	B(OH)_2_	Pd_2_(dba)_3_ (2.5 mol %), TFP (7.5 mol %), CuTC (3 equiv.), THF, 55 °C	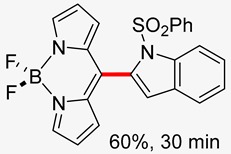

**Table 11:** Transition-metal-catalyzed reactions of indolylboronic acids with alkenes.

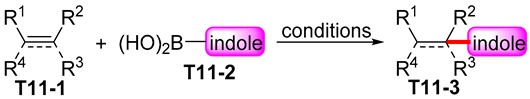			
**Entry**	**T11-1**	**Conditions**	**T11-3**
1	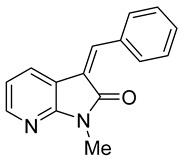	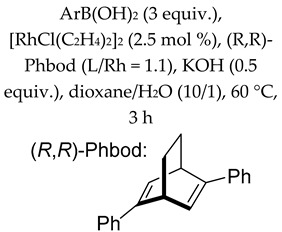	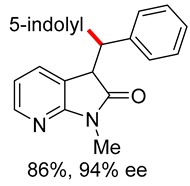
2	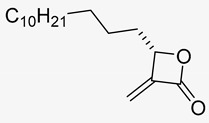	ArB(OH)_2_ (1.5 equiv.), [Rh(cod)Cl]_2_ (1 mol %), KOH (1 equiv.), dioxane/H_2_O (10:1), 60 °C, 1 h	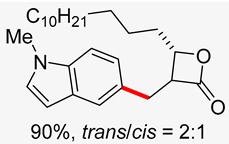
3	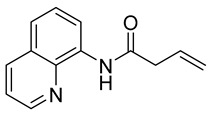	ArB(OH)_2_ (2 equiv.), Pd(OAc)_2_ (10 mol %), NaF (2 equiv.), TFT, 100 °C, 12 h	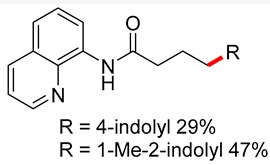
4	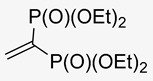	ArB(OH)_2_ (1.5 equiv.), Cu(OTf)_2_ (10 mol %), toluene, 70 °C, 18 h	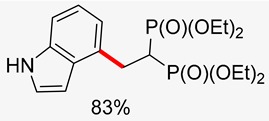
5	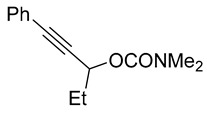	ArB(OH)_2_ (2.5 equiv.), Ni(cod)_2_ (10 mol %), TrixiePhos (10 mol %), THF (0.05 M), rt, 24 h